# Mechanistic insights into metronidazole activation and antiprotozoal action in anaerobic parasites

**DOI:** 10.3389/fpara.2026.1946584

**Published:** 2026-10-13

**Authors:** Anwesha Bandyopadhyay, Sudip K. Ghosh

**Affiliations:** Department of Bioscience and Biotechnology, Indian Institute of Technology Kharagpur, Kharagpur, India

**Keywords:** cell death, *Entamoeba*, *Giardia*, *Helicobacter*, metronidazole resistance, *Trichomonas*

## Abstract

Metronidazole (MTZ) is a nitroimidazole compound widely used as a drug for the treatment of infections caused by anaerobic and microaerophilic pathogens, including *Entamoeba histolytica*, *Giardia lamblia*, *Trichomonas vaginalis*, and *Helicobacter pylori*. The drug is administered as a prodrug that requires intracellular reduction to generate cytotoxic nitro radicals that damage DNA and other cellular macromolecules. Four major and an additional but overlapping pathway exclusively in *Trichomonas* have been described. In protozoan parasites *Entamoeba*, *Giardia*, and *Trichomonas*, resistance is often associated with alterations in PFOR and ferredoxin-mediated metabolic pathways responsible for drug activation. Studies have shown that enzymes such as oxygen-insensitive nitroreductases (NTRs) activate MTZ, whereas nitroimidazole reductases (NIMs) can convert the drug into non-toxic derivatives, thereby contributing to resistance phenotypes in *Entamoeba*. Recent advances indicate the activation of an alternative pathway involving MTZ reduction, and activation by the malic enzyme. Additionally, transcriptomic analyses of resistant *Giardia* lines reveal changes in the redox metabolism, oxidative stress response, and energy metabolism that may reduce drug activation or detoxify reactive intermediates.Recently, our research team has reported that the MTZ-associated oxidative stress induces the activation of the Apoptosis-inducing Factor, and nucleophagy, which, together, contribute to the trophozoite death in *E. histolytica*. Effective treatment strategies are also significantly intensifying, with increasing reports of MTZ resistance. On the other hand, in *Helicobacter pylori*, resistance frequently arises through mutations or inactivation of nitroreductase-encoding genes such as *rdxA* and *frxA*, which prevent the conversion of MTZ into its active form. Other mechanisms, including increased oxidative stress and cellular efflux systems, may also contribute to reduced susceptibility. Overall, anaerobic and microaerophilic pathogens are involved in the continuous process of developing newer strategies to defeat the antimicrobial activity of MTZ as a part of co-evolution. Understanding these molecular mechanisms is essential for improving therapeutic strategies and finding new drug targets as well as developing new nitroimidazole derivatives or combination therapies. Therefore, we attempt to make a comprehensive review on the mechanisms of MTZ activation, cell death mode(s) triggered by MTZ, as well as the pathogenic strategies to develop resistance against this drug, including all the recent advances.

## Graphical Abstract

**Figure:**
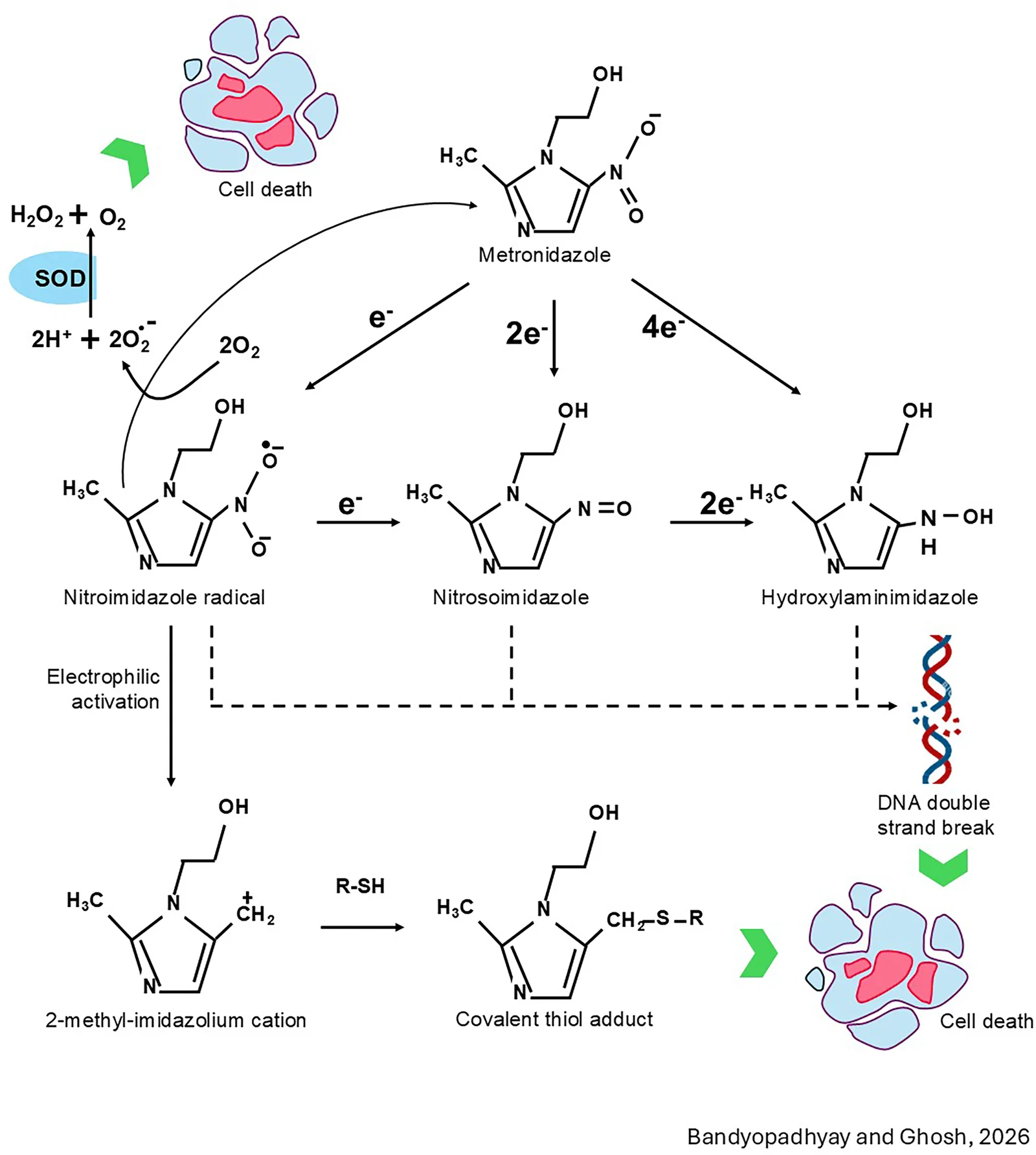
Mechanism of metronidazole activation and cytotoxicity in anaerobic protozoan parasites and bacteria. Intracellular reduction of metronidazole generates reactive nitroimidazole intermediates that induce DNA damage and form covalent thiol adducts, disrupting redox homeostasis, leading to parasite death. Superoxide dismutase (SOD) converts the nitroimidazole radical back into metronidazole, with H_2_O_2_ as a byproduct, which may synergistically kill the parasites.

## 1 Introduction

Metronidazole (MTZ), or 2-methyl-5-nitroimidazol-1-ethanol, is an FDA-approved drug, first used for the treatment of trichomoniasis, and later expanded to the treatment of anaerobic protozoal infections such as amoebiasis, giardiasis, and anaerobic bacterial infections (Samuelson, 1999). MTZ has a high bioavailability with satisfactory tissue distribution and low serum protein binding property (Lau et al., 1992). It is administered in a prodrug form that is uptaken by cells through diffusion, followed by intracellular reduction to an active form which interacts with DNA, disrupts its helical structure, and introduces breaks in nucleic acid strands, eventually leading to cell death (Weir and Le, 2023). Due to its intrinsically low redox potential, MTZ readily undergoes reduction within cells. This reduction is catalyzed by intracellular reductase enzymes whose own redox potentials are sufficiently low to facilitate electron transfer to MTZ, and depending on the number of electrons transferred to its nitro group, different types of intermediates are formed. Serial transfer of one, two and four electrons produces a nitroimidazole radical anion, a nitrosoimidazole and a hydroxylamineimidazole intermediate, one by one. Accordingly, a one-step direct transfer can only produce the hydroxylamineimidazole intermediate (Leitsch, 2019) (**Figure 1**). These intermediates, besides binding with DNA, have high reactive potential towards protein sulfhydryl (-SH) groups of strong nucleophiles, particularly thiol proteins, forming covalent thiol adducts (**Figure 1**), ultimately resulting in cytotoxicity in susceptible cells (Müller, 1986).

**Figure 1:**
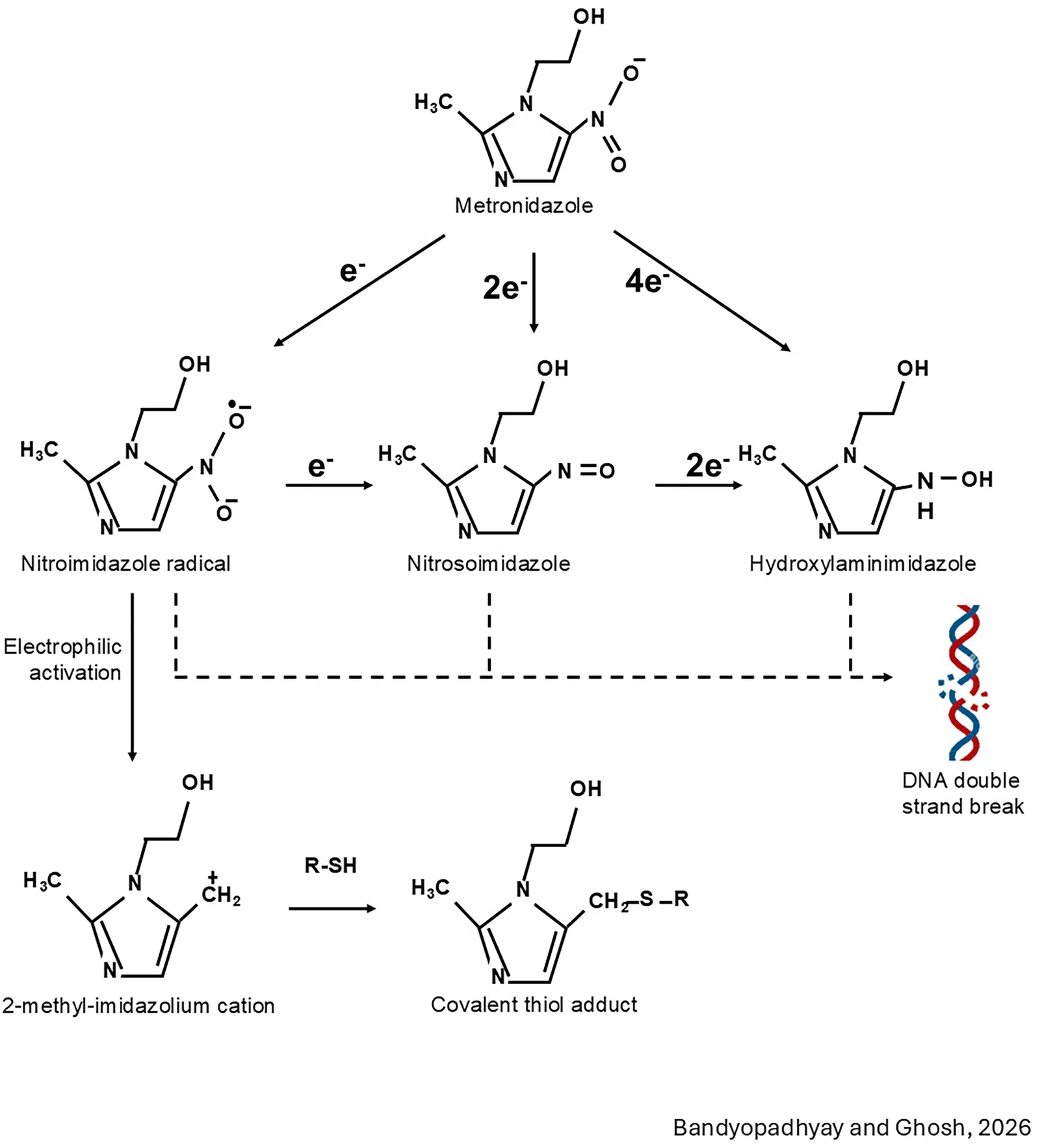
Reduction of metronidazole. Transfer of one electron, two electrons, and four electrons generate nitroimidazole radical, nitrosoimidazole radical, and hydroxylaminimidazole intermediates respectively. Transfer of more than one electron can be direct or sequential depending on mostly organismal preference. All three intermediates are capable of binding to DNA and double strand breakage. Nitroimidazole can undergo electrophilic activation to form 2-methyl-imidazolium cation which eventually binds and inactivates protein and non-protein thiol molecule by forming covalent thiol adducts.

The hydroxylamineimidazole intermediate is the primary DNA-damaging species and is produced by the 4-electron reduction of the nitro group during MTZ activation. It is stable enough to diffuse from the site of reduction to reach DNA and other cellular targets, and reactive enough to form covalent adducts with the targets. It can undergo further reactions to generate even more reactive species. Its lethal effects are mediated through three interconnected mechanisms:

### 1.1 DNA damage and genotoxic stress

Hydroxylamineimidazole undergoes protonation and dehydration to form a reactive nitrenium ion (R – NH^+^) that makes covalent bonds with DNA bases, prevalently with the guanine N7 and N2 positions. Formation of these adducts distorts the DNA double helix, resulting in mispairing, replication fork stalling, and transcription blocking. When the resulting single-strand breaks occur in the close proximity of opposing strands, they generate double-strand breaks (directly demonstrated in *Giardia* by Ordoñez-Quiroz et al., 2018). This leads to the cell-cycle arrest in the S-phase. Depending on the organism, the DNA damage response may be activated, as evidenced by H2A phosphorylation and attempts to repair by homologous recombination (Uzlikova and Nohynkova, 2014). If damage outweighs repair capacity, the cell cycle arrest becomes irreversible, and death pathways are initiated.

### 1.2 Protein adducts formation followed by redox catastrophe

Hydroxylamineimidazole forms covalent S – N bonds with cysteine residues of a limited but functionally significant set of proteins, forming adducts. A notable target is thioredoxin reductase, whose MTZ adduct destroys its enzymatic activity, thereby compromising the thioredoxin/peroxiredoxin antioxidant system, the primary defence against oxidative stress in the target organisms (Leitsch et al., 2007). This eventually establishes a self-amplifying cycle; drug activation inactivates the enzyme that protects against drug-induced oxidative damage, leading to progressive accumulation of reactive oxygen species. In *E. histolytica*, five major protein targets by MTZ for adduct formation were identified, which are thioredoxin, thioredoxin reductase, superoxide dismutase, purine nucleoside phosphorylase, and one uncharacterized protein (Leitsch et al., 2007). Cysteine supplementation was found to rescue from oxidative damage, demonstrating that thiol depletion can be linked to death. In *T. vaginalis*, seven proteins form adducts, including malate dehydrogenase, disrupting energy metabolism) and ribonucleotide reductase, interfering with DNA synthesis (Leitsch et al., 2009).

### 1.3 Metabolic collapse and terminal execution

The combined effects of DNA damage and protein inactivation lead to the progressive metabolic collapse with substantial ATP depletion, loss of ion homeostasis, membrane depolarization, and restricted protein synthesis. Depending upon the concerned organism, the redox imbalance triggers different predominant pathways, like lipid peroxidation and glycerophospholipid disruption in *Giardia* (Popruk et al., 2023; Raj et al., 2024), membrane rupture, organelle destruction, and global transcriptional shutdown in *T. vaginalis* (Xie et al., 2023), and AIF activation in *E. histolytica* (Bandyopadhyay and Ghosh, 2023a).

The nitro radical anion, the nitroso derivatives and the reactive oxygen species (ROS) are highly electrophilic. They attack the phosphodiester backbone of DNA, causing single- and double-strand breaks, loss of helical structure (Edwards, 1993). Nitroso groups are potent thiol-scavenging agents, readily reacting with thiols (-SH) of glutathione or cysteine residues to form semimercaptals, sulfinamides, or disulfides (Noss et al., 1988) and perturbs the redox homeostasis of thiol-dependent anaerobic protozoa. This group can also serve as a ligand for transition metals. It can chelate iron-sulfur clusters present in ferredoxin and PFOR, thereby destroying the catalytic core of the electron transport chain and halting ATP production (McClelland et al., 1987). 2-Methylimidazolium cation derivatives undergo rapid reduction, followed by oxidation by intracellular oxygen species, triggering a lethal burst of oxidative stress via superoxide and hydrogen peroxide through redox-cycling.

The positive charge on the imidazolium ring allows the molecule to form strong electrostatic interactions with the negatively charged phosphate groups of DNA and position the reactive nitro group adjacent to the DNA helix, thereby ensuring adduct formation (Biaglow et al., 1986). MTZ is particularly active against anaerobic protozoans that possess low redox electron transport proteins like ferredoxin that is capable of activating the drug inside the organism. The classically sensitive protozoans to MTZ are *Entamoeba histolytica*, *Giardia lamblia*, and *Trichomonas vaginalis* whereas *Balantidium coli*, and *Dientamoeba fragilis* are also documented to be responsive towards MTZ (Schuster and Ramirez-Avila, 2008; Gotfred-Rasmussen et al., 2021). Amongst bacteria, some gram-positive (e.g. *Actinomyces*) and gram negative (e.g. *Porphyromonas*, *Velionella*) anaerobes are also MTZ sensitive (**Table 1**). *Helicobacter pylori* is a microaerophilic gram-negative bacterium that is MTZ sensitive, and therefore MTZ is widely used for pylorisis treatment as well. In this review, we will discuss mechanistic insights into MTZ activation and resistance in *E. histolytica*, *G. lamblia*, and *T. vaginalis* as protozoal pathogens, and in *H. pylori* as a bacterial pathogen, given their significant clinical significance. It is worth noting that most of our understanding of MTZ resistance in anaerobic protozoa and bacteria is derived from laboratory-selected resistant strains, which may not fully reflect the real-world mechanisms of resistance, i.e. in clinical isolates. Where possible, we have distinguished among clinical, laboratory-derived, and experimental evidence and highlighted mechanisms validated in clinical settings.

**Table 1:** A list of metronidazole sensitive pathogenic bacteria and unicellular protozoan parasites (Freeman et al., 1997).

Category	Organism/group	Clinical relevance/notes on metronidazole susceptibility
Gram-negative anaerobic bacteria	*Bacteroides fragilis* group	Commonly susceptible; important anaerobic pathogens in intra-abdominal and mixed infections
	*Prevotella* spp.	Frequently susceptible; associated with oral, respiratory, and genital tract infections
	*Porphyromonas* spp.	Usually susceptible; important in periodontal and oral infections
	*Fusobacterium* spp.	Generally susceptible; implicated in oral, head–neck, and systemic anaerobic infections
	*Veillonella* spp.	Usually susceptible anaerobic cocci; part of polymicrobial infections
Gram-positive anaerobic bacteria	*Clostridioides* (*Clostridium*) spp.	Important anaerobic pathogens; metronidazole active against several species
	*Peptostreptococcus* spp.	Commonly susceptible; involved in mixed anaerobic infections
	*Peptococcus* spp.	Generally susceptible anaerobic cocci
	*Actinomyces* spp.	Susceptibility variable; metronidazole activity less predictable
Microaerophilic bacteria	*Helicobacter pylori*	Included as part of combination therapy rather than used alone
Anaerobic protozoa (classically sensitive)	*Entamoeba histolytica*	Highly susceptible; causes amoebiasis (intestinal and extra-intestinal, including liver abscess)
	*Giardia lamblia (G. duodenalis)*	Highly susceptible; causes giardiasis
	*Trichomonas vaginalis*	Highly susceptible; causes trichomoniasis
Anaerobic protozoa (also responsive)	*Balantidium coli*	Responsive; associated with balantidiasis
	*Dientamoeba fragilis*	Response documented in intestinal protozoal infection
Anaerobic protozoa (variable response)	*Blastocystis* spp.	Susceptibility inconsistent; metronidazole may or may not be effective

### 1.4 Trichomonas vaginalis

*T. vaginalis* is a flagellated protozoan parasite that infects the urogenital tract and causes trichomoniasis, one of the most common nonviral sexually transmitted infections worldwide. Infection is transmitted primarily through sexual contact, with trophozoites colonizing the vagina, urethra, and paraurethral glands in females and the urethra and prostate in males. Direct physical contact is a prerequisite for transmission of the pathogen, since *T. vaginalis* exists only in the trophozoite form and without any cyst stage. The parasite attaches firmly to the urogenital epithelium by means of surface adhesins, releases proteolytic enzymes to degrade host tissue and extracellular matrix, lyses epithelial cells by contact-dependent damage, perturbs vaginal pH and normal flora, and finally raises a tissue-damaging proinflammatory immune response in the host. The manifestation of common vaginitis symptoms in the infected hosts raises concerns because of an increased risk of adverse pregnancy outcomes and HIV secondary infection in *Trichomonas*-infected individuals (Mercer and Johnson, 2018). The recommended treatment for trichomoniasis is a single dose (2 g) of MTZ for both the index patient and their sexual partners. After single-dose treatment, high rates of retest positivity are reported, which are probably caused by clinical resistance rather than medication resistance or re-infection (Kissinger, 2015). The protoplasm of *Trichomonas* contains a very specialized double membrane-bound organelle with electron-dense granular matrix called hydrogenosome. Hydrogenosome is primarily involved in anaerobic energy metabolism and therefore houses a number of enzymes essential for *Trichomonas* survival (Benchimol et al., 1996).

### 1.5 Giardia lamblia

*G. lamblia* (also known as *G. intestinalis* or *G. duodenalis*) is a flagellated protozoan parasite that infects the small intestine and causes giardiasis, one of the most common waterborne diarrheal diseases worldwide. Here also, the infection begins with the ingestion of cysts through contaminated water or food. After exposure to gastric acid, excystation occurs in the duodenum, releasing trophozoites that colonize the upper small intestine. Unlike *Entamoeba*, Giardia does not penetrate the intestinal mucosa; rather, it attaches to the mucosal surface with an adhesive disc, interfering with nutrient absorption (Adam, 2001). Disruption of the intestinal epithelium alters intestinal permeability, leading to fluid secretion in the lumen. Microvillus shortening causes lactase-like enzyme deficiency in the brush-border. Together, all these lead to nutrient malabsorption, fluid secretion and dysbiosis (Ankarklev et al., 2010). Classical therapy of *G. lamblia* infection primarily involves nitroimidazole drugs such as MTZ, which disrupts the parasite’s DNA and are usually administered for 5–7 days. Alternative agents like tinidazole (single-dose therapy) and nitazoxanide are also effective, particularly in cases of intolerance or resistance (Gardner and Hill, 2001).

### 1.6 Entamoeba histolytica

*E. histolytica* is a potent pathogen causing the disease amoebiasis, which is the second leading cause of death from parasitic infection. *Entamoeba* is a unicellular anaerobic protozoan parasite having a biphasic life cycle comprising an invasive trophozoite and an infective cyst stage. Infection of the host body occurs through the ingestion of cyst-contaminated food or water that germinates into trophozoites inside the host’s GI tract. Trophozoites inhabit the colonic lumen and mucosa, causing amoebic colitis. Sometimes, it may cross the intestinal barrier and be carried through the circulation to ectopic locations like the liver, lungs and brain, resulting in abscesses, which may turn fatal if left untreated. *E. histolytica* phagocytose red blood cells, secretes several virulence factors including Gal/GalNAc lectin, amoebapore and proteinases that destroy host tissues (Zhang et al., 2026; Faust and Guillen, 2012). MTZ is the primary choice for amoebiasis treatment for decades, though concerns are rising gradually with the increasing case reports on MTZ unresponsiveness (Singh et al., 2023).

However, it is generally found that key enzymes to reduce and activate MTZ in anaerobic unicellular protozoans are Pyruvate:ferredoxin oxidoreductase (PFOR) and Nitroreductase.

### 1.7 Helicobacter pylori

*H. pylori* is a spiral-shaped, microaerophilic, Gram-negative bacterium that colonizes the human stomach and is strongly associated with chronic gastritis, peptic ulcer disease, and gastric malignancies. *H. pylori* employs several kinds of virulence mechanisms to establish a persistent infection and is particularly adapted to survive in the acidic stomach environment. The bacterium produces urease in abundance, which hydrolyses urea into carbon dioxide and ammonia, thereby neutralizing stomach acid and providing a safe environment. Motility and colonization at the epithelial surface of the gastric mucus layer are enabled by its flagella followed by adhesion to epithelial cells by means of adhesins, such as BabA. As a professional pathogen, it secretes a number of virulence factors, including the cytotoxin CagA (cytotoxin-associated gene A), which is injected into host cells via a type IV secretion system, causing inflammation, altered cell signalling, and enhanced carcinogenic potential. Another notable virulence factor is vacuolating cytotoxin A (VacA), which mediates apoptosis, vacuolation, and cell damage (Kusters et al., 2006). Clinical outcomes of pylorisis include gastritis, peptic ulcers with nausea, bloating, and epigastric pain. Chronic gastritis can lead to gastric adenocarcinoma and MALT lymphoma (IARC Working Group on the Evaluation of Carcinogenic Risks to Humans, 1994). Treatment of *Helicobacter* infection also includes MTZ, amoxicillin, bismuth, and acid-suppression medication.

## 2 Mechanistic insights into the Pyruvate:ferredoxin oxidoreductase-mediated activation of metronidazole

PFOR is a key enzyme in anaerobic energy metabolism that catalyzes the oxidative decarboxylation of pyruvate to acetyl-CoA while transferring electrons to ferredoxin. Lactate dehydrogenase and pyruvate decarboxylase, principal fermentation enzymes found in yeast and other eukaryotes, are absent in amoebae and *Giardia*. Instead, they acquired bacterial fermentation enzymes, including PFOR, an iron-sulphur protein which is involved in MTZ activation. Reduced ferredoxin, generated by the enzymatic activity of PFOR, donates electrons to NAD in the case of amoeba, *Giardia* and to protons in the case of trichomonads. The ferredoxins of amoeba and *Giardia* resemble bacterial ferredoxin (6 kD), whereas the ferredoxins of trichomonads resemble mitochondrial ferredoxin (Samuelson, 1999).

In anaerobic protozoa such as *Entamoeba histolytica*, *Giardia lamblia*, and *Trichomonas vaginalis*, the reduced ferredoxin generated by PFOR serves as a major electron donor for the one-electron activation of MTZ, producing cytotoxic radical intermediates. Consequently, decreased PFOR activity or impaired electron transfer to ferredoxin is associated with reduced drug activation and contributes to MTZ resistance. Thus, PFOR plays a central role linking core metabolism to nitroimidazole susceptibility in anaerobic pathogens (Müller, 1983).

### 2.1 T. vaginalis

As discussed above, MTZ is administered as a prodrug and is inactive until reduced by the parasites’ anaerobic/microaerophilic metabolic enzymes. Uptake of MTZ by *T. vaginalis* and enzymatic removal of its 5-nitro group within the cell were recorded in 1974 by Ings et al. (1974). Formation of DNA and protein adducts, but not RNA adducts, with MTZ derivatives was also demonstrated in this study by ^14^C radioactive incorporation and subsequent inhibition of nucleic acid synthesis. The PFOR in *Trichomonas* is a hydrogenosomal membrane-bound enzyme (Williams et al., 1987) that donates an electron to MTZ, through ferredoxin, generating a cytotoxic free radical (**Figure 2A**). The 60% reduction in the abundance of the intracellular PFOR transcript in the MTZ-resistant *T. foetus* indicates its essential, primary role in the MTZ activation pathway. In addition to PFOR, the hydrogenase and ferredoxin also showed 90-98% reduction in their mRNA levels in the MTZ-resistant strain compared to the sensitive ones (Land et al., 2001), suggesting that these enzymes/factors are also inextricably associated with the MTZ’s antiparasitic activity. Crossnoe et al. (2002) determined the three-dimensional structure of the *T. vaginalis* hydrogenosomal oxidized ferredoxin [2Fe-2S] with nine hydrogen bonds connecting to the sulphur atoms of the cluster, a fold similar to other ferredoxins, and a unique cavity adjacent to the iron-sulphur cluster exposing one inorganic sulphur atom to the solvent, rendering it highly reductive in nature, triggering MTZ activation. However, it is noteworthy that Hydrogenase accepts electrons from reduced ferredoxin to produce molecular hydrogen (H_2_). MTZ competes for the same pool of low-potential electrons, and since fewer electrons flow through the hydrogenosomal pathway in the presence of a functional hydrogenase, less MTZ is reduced. Thus, hydrogenase may contribute indirectly to drug susceptibility by maintaining the electron-transfer environment necessary for MTZ activation (Dunne et al., 2003). Although hydrogenase itself does not chemically activate MTZ, when available inside the cell, MTZ ‘hijacks’ and diverts these electrons before hydrogenase can use them. Therefore, hydrogenase determines the availability and flow of low-redox-potential electrons, making it an indirect but essential participant in MTZ activation (Jaromin et al., 2025).

**Figure 2:**
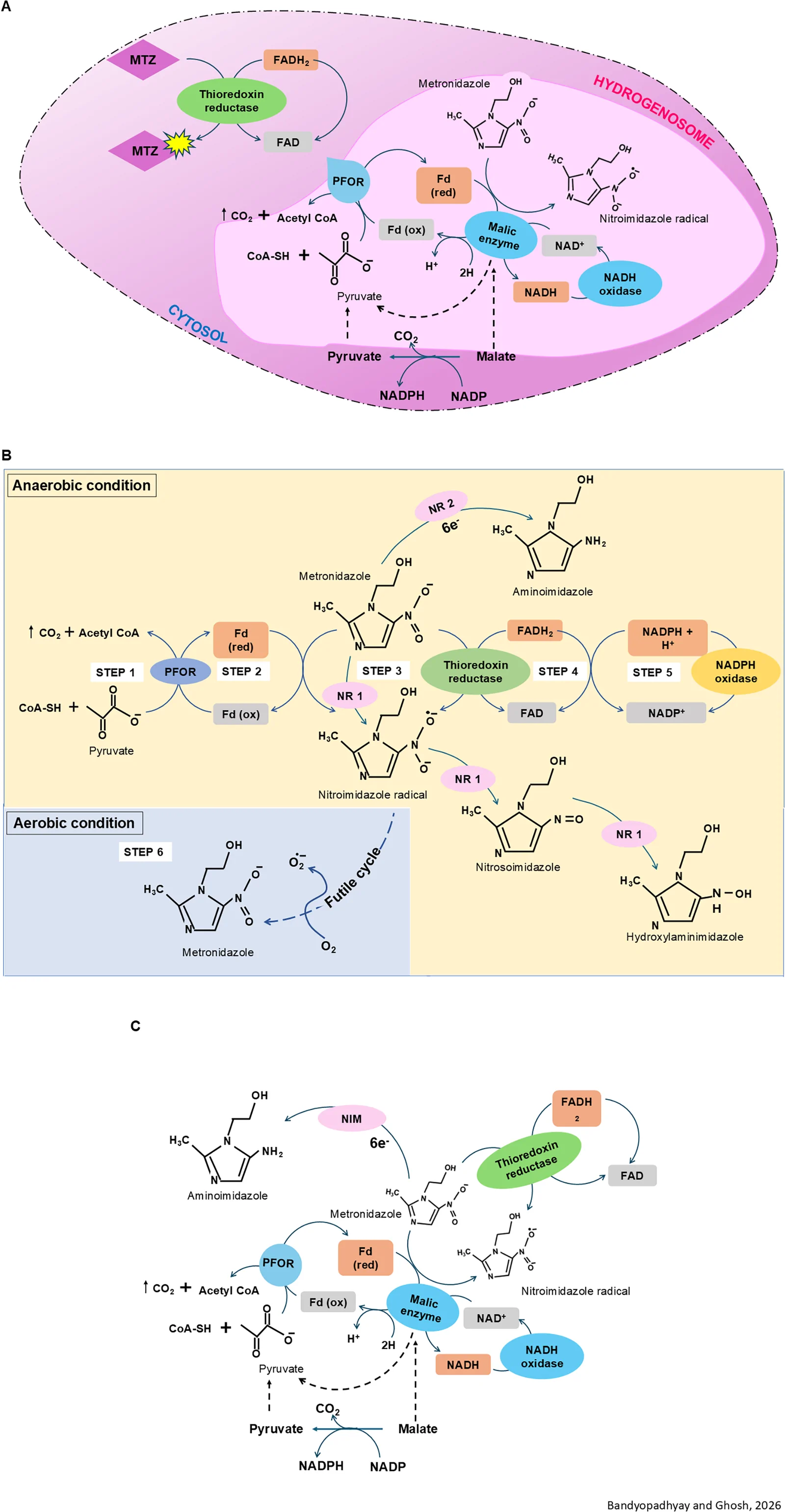
Metronidazole activation pathways in **(A)** *Trichomonas vaginalis*. Three major pathways involved in the MTZ reduction and activation are the PFOR-mediated, malic enzyme-mediated, and thioredoxin reductase-mediated. The PFOR and malic enzyme reduces MTZ within the hydrogenosome, the specialized organelle in *Trichomonas*, whereas the thioredoxin reductase reduces MTZ in the cytosol. **(B)** *Giardia lamblia.* In anaerobic conditions, MTZ is reduced by PFOR and thioredoxin reductase in the cytosol of *Giardia*, whereas under aerobic conditions, metronidazole undergoes a futile cycle in which an oxygen molecule accepts an electron from the nitroimidazole, which then converts back to metronidazole. The *Giardia* nitroreductase 1 performs one-step and serial electron donation to MTZ, generating all types of intermediates. The nitroreductase 2 catalyses a one-step reduction of MTZ by donating six electrons at a time to form non-reactive aminoimidazole compound. **(C)** *Entamoeba histolytica.* In anaerobic conditions, MTZ is reduced via three major pathways, catalysed by PFOR, malic enzyme, or thioredoxin reductase in the cytoplasm. NIM enzyme inactivates MTZ by producing inactive aminoimidazole compound through a one-step reduction with six electrons. MTZ, metronidazole; Fd, ferredoxin; PFOR, pyruvate-ferredoxin oxidoreductase; NR, nitroreductase; NIM, nitroimidazole reductase.

### 2.2 G. lamblia

Similar to *Trichomonas*, the primary pathway of MTZ reduction in *Giardia* involves PFOR that transfers electrons from pyruvate to ferredoxin (Fd). Unlike *Trichomonas*, *Giardia* lacks a hydrogenosome; therefore, MTZ reduction occurs in the cytosol. Townson et al. (1996) characterized and purified functional PFOR from *G. duodenalis* and experimentally demonstrated that no pyridine nucleotide (NAD^+^ or NADP^+^) participates in the catalytic cycle. Instead, reduced ferredoxin subsequently donates electrons for processes such as MTZ activation. Like ferredoxin, this pyruvate oxidation in *Giardia* is also catalyzed by a eubacterial-like PFOR where electrons are transferred to ferredoxin rather than NAD^+^ (Brown et al., 1998). Three ferredoxins were identified in *Giardia*, of which only Fd I was demonstrated to have an iron-sulphur centre and to serve as an electron acceptor from pyruvate dehydrogenase, and can utilize MTZ as an electron acceptor. The other two ferredoxins (Fd II and Fd III) did not accept electrons from PFOR, indicating that they are not coupled to the PFOR-dependent MTZ activation pathway (Townson et al., 1996). The reduction of the nitro group of MTZ renders the drug ‘active’ (**Figure 2B**; step 3). This chemical modification of MTZ creates a favourable gradient, allowing further drug uptake by the parasite. Although MTZ was effective against *Giardia* trophozoites, as evident from reduced respiration and motility, a notable impact on cysts was not observed (Paget et al., 1989), probably due to the poor penetration through the Gal/GalNAc lectin polymer of the chitin wall of the cyst (Thompson et al., 1993).

### 2.3 E. histolytica

*Giardia* and *Entamoeba* are intestinal protozoan parasites that lack classical mitochondria and a hydrogenosome-analogue organelle, have independently adapted to anaerobic environments and therefore share several metabolic features, including reliance on PFOR, ferredoxin-mediated electron transfer, and susceptibility to nitroimidazole drugs. Electrons are transferred from pyruvate to MTZ through ferredoxin by PFOR (Duchêne, 2016). PFOR is the central enzyme linking glycolysis to anaerobic energy metabolism. Classical pyruvate dehydrogenase is absent in *Entamoeba*; thus, by governing the oxidative decarboxylation of pyruvate to synthesize acetyl-CoA, PFOR replaces the dependence of *Entamoeba* on NAD^+^ that normally forms a complex with the pyruvate dehydrogenase (**Figure 2C**). Unlike in *T. vaginalis* and some laboratory-selected *Giardia* strains, PFOR expression is not consistently reduced in MTZ-resistant *E. histolytica*. Resistance in *Entamoeba* appears to involve a broader network of redox adaptations, including changes in antioxidant enzymes such as superoxide dismutase, peroxiredoxin, and flavin reductase, rather than simple downregulation of PFOR alone (Duchêne, 2016).

## 3 Mechanistic insights into the malic enzyme-mediated activation of metronidazole

In anaerobic and microaerophilic protozoan parasites, the NAD(P)-dependent malic enzyme (ME) is of great significance at the interface between carbon metabolism and intracellular electron transfer. ME catalyzes the oxidative decarboxylation of L-malate to pyruvate, generating NADH or NADPH (depending on the isoform) and CO_2_. The resulting pyruvate can subsequently be metabolized by PFOR, which transfers electrons to ferredoxin, thereby linking malate metabolism to the low-redox-potential electron transport pathways characteristic of anaerobic protists. These electrons ultimately participate in energy conservation and, in parasites such as *Giardia*, *Entamoeba*, and *Trichomonas*, contribute indirectly to the reductive activation of nitroimidazole drugs through the PFOR-ferredoxin pathway (Müller, 1988; Brown et al., 1998; Adam, 2001). In addition to supplying pyruvate, malic enzyme (ME) contributes to cellular redox homeostasis by producing NADPH, which fuels antioxidant systems including thioredoxin- and peroxiredoxin-dependent pathways. Thus, ME supports both metabolic flux and protection against oxidative stress. In *E. histolytica*, ME forms part of the central fermentative metabolism together with PFOR and other acetyl-CoA-synthesizing enzymes, whereas in *G. duodenalis*, the enzyme complements the bacterial-type anaerobic metabolic network that replaces the conventional mitochondrial pyruvate dehydrogenase complex (Brown et al., 1998; Adam, 2001; Duchêne, 2016). Although ME is not a direct electron carrier, its reducing equivalents and the ability to generate pyruvate make it an important upstream contributor to electron flow through the PFOR-ferredoxin pathway, thereby indirectly influencing the efficiency of anaerobic energy metabolism and nitroimidazole activation.

### 3.1 T. vaginalis

In the study by Land et al. (2001), along with the downregulation of PFOR, hydrogenase and ferredoxin, interestingly, the malic enzyme mRNA level was also approximately 85% less in the MTZ-resistant strain than in the sensitive strain. This study demonstrates that ME is downregulated alongside PFOR, hydrogenase, ferredoxin, and other hydrogenosomal enzymes, and concludes that high-level MTZ resistance results from extensive remodelling or functional reduction of hydrogenosomal metabolism rather than alterations in a single component.

Following this, the MTZ sensitivity of the PFOR-deficient *T. vaginalis* strain (TV 10–02 MR 5) further strengthened the hypothesis of an alternative MTZ activation pathway, ultimately leading to the discovery of malic enzyme-mediated reduction of MTZ in the hydrogenosome. Electrons originate from malate oxidation by NAD-dependent malic enzyme, which transfers them through NADH:ferredoxin oxidoreductase to ferredoxin. This is an additional MTZ reduction pathway in hydrogenosomes (Hrdý et al., 2005). The malic enzyme, another hydrogenosomal enzyme, oxidatively decarboxylate malate, and transfers an electron from malate to NAD^+^ (**Figure 2A**). The resulting NADH further transfers an electron to ferredoxin by NADH dehydrogenase (Hrdý et al., 2005). The reaction is as follows-

**Table:** 

Step 1: Oxidative decarboxylation of malate	Malate + NAD^+^ → Pyruvate + CO_2_ + NADH + H^+^
Step 2: Electron transfer from NADH to ferredoxin	NAD^+^ + Fd(red) ← Fd(ox)

The reduced ferredoxin, thereafter, transfers the electron to MTZ similar to the conventional pathway. This is direct biochemical evidence suggesting that ME is necessary for this pathway, but does not itself reduce MTZ, nevertherless, explains the MTZ resistance due to the loss of ME. Since ME provides pyruvate to PFOR, reduced ME expression limits the availability of substrate for the PFOR-ferredoxin pathway. Consequently, fewer low-redox-potential electrons are generated for the reductive activation of MTZ, contributing to the high-level drug resistance. In resistant strains, the downregulation of ME mRNA directly correlates with the previous observation (Land et al., 2001) though there is no direct evidence to support that ME downregulation alone can confer resistance against MTZ in *T. vaginalis*.

### 3.2 E. histolytica

Similar to *Trichomonas*, an alternative pathway of MTZ reduction in *Entamoeba* by malic enzyme (EhME) has also been implicated. Treatment of *E. histolytica* trophozoites with MTZ increased EhME mRNA and protein levels (approximately 2.1-fold at lower drug concentrations), suggesting that the parasite upregulates malic enzyme in response to MTZ exposure in a dose-dependent manner, potentially reinforcing its own drug activation pathway (Chakrabarty et al., 2025). Upregulation of EhME may be attributed to correlative evidence since upregulation could be a stress response, not necessarily related to drug activation, and therefore, additional experiment-based evidence is needed to confirm direct ME-mediated activation of MTZ. Crystal structure determination revealed that EhME forms a unique, deeply interlocked dimer resembling bacterial malic enzymes. Although this structural feature is not directly responsible for electron transfer, it may underlie the enzyme’s catalytic properties and evolutionary conservation. The authors propose that malic enzyme-mediated activation helps explain why loss or reduction of previously identified reductases does not always produce high-level MTZ resistance. EhME may therefore function alongside the established PFOR-ferredoxin pathway to sustain drug activation. EhME is an NADP^+^-specific enzyme, but unlike competitively inhibitable *Trichomonas* ME, it is inhibited by ATP non-competitively with NADP^+^ and uncompetitively with L-malate, thereby creating feedback regulation in the ME-dependent metabolic pathways. Jeelani et al. (2010) indicated that two NADPH-dependent oxidoreductases, EhNO1 and EhNO2, which are involved in ferric ion and cysteine reduction respectively, could also be potent activators of MTZ. MTZ activation pathways in *E. histolytica* are diagrammatically represented in **Figure 2C**.

Although the ME has been well implicated in MTZ activation in both *Trichomonas* and *Entamoeba*, no direct or indirect evidence of ME involvement has been found to date in *Giardia*. *Giardia* genome may house more than one copy of malic enzyme (GiardiaDB), and therefore, it opens a window of research opportunity to study the role, if any, of malic enzyme in MTZ activation pathway in *Giardia*, their structural similarities with ME of *Trichomonas* and *Entamoeba*, and its potential to be a drug target.

## 4 Mechanistic insights into the thioredoxin reductase-mediated activation of MTZ

Thioredoxin reductase (TrxR) is a flavoprotein oxidoreductase that plays a central role in maintaining intracellular redox homeostasis in anaerobic protozoan parasites. Using NADPH as an electron donor, TrxR reduces oxidized thioredoxin, which subsequently transfers electrons to a variety of target proteins involved in DNA synthesis, antioxidant defense, and peroxide detoxification. In parasites such as *Giardia*, *Entamoeba*, and *Trichomonas*, the thioredoxin system compensates for the absence of glutathione-dependent redox pathways and serves as the primary mechanism of protection against oxidative stress (Müller et al., 2003; Leitsch et al., 2007). Beyond its physiological role, TrxR has emerged as an important enzyme in nitroimidazole metabolism. In *E. histolytica*, TrxR primarily functions in antioxidant defense by reducing thioredoxin, which subsequently donates electrons to peroxiredoxin and other thiol-dependent enzymes, thereby maintaining redox balance under oxidative stress (Leitsch et al., 2007). Consequently, thioredoxin reductase serves both as a central electron transfer enzyme in protozoan redox metabolism and, in certain species, as an alternative activator of nitroimidazole drugs.

### 4.1 T. vaginalis

Depletion of intracellular iron, with minimal expression of PFOR and malate dehydrogenase (hydrogenosomal) in *Trichomonas*, did not reduce MTZ susceptibility. The first successful targeted gene replacement of ferredoxin 1 in *T. vaginalis* (Land et al., 2004) did not confer resistance to MTZ either, ruling out the possibility that ferredoxin-mediated pathways are the only critical determinants of MTZ activation or resistance. Leitsch et al. (2009) purified recombinant thioredoxin reductase (TrxR) from *T. vaginalis* and showed that it can directly reduce MTZ as well as other 5-nitroimidazole drugs using NADPH as an electron donor (**Figure 2A**). By a comparative proteomic analysis between MTZ-treated and untreated *T. vaginalis* cell lysates, the authors identified seven proteins that form covalent adducts with MTZ-derived reactive intermediates by proteomic analysis of MTZ-treated *T. vaginalis* cell lysates, including Malate dehydrogenase, Ribonucleotide reductase, and Thioredoxin reductase. The thioredoxin-reducing activity of TrxR was significantly impaired due to the adduct formation, suggesting that TrxR not only activates the drug but also becomes a target of its own activation products (Leitsch et al., 2009). Notably, no hydrogenosomal proteins (including PFOR or ferredoxin) were found among the adducted proteins, suggesting that in *T. vaginalis*, the cytosolic TrxR pathway may be more physiologically relevant for drug activation than the hydrogenosomal PFOR/ferredoxin pathway, at least in terms of the spatial relationship between the site of toxic intermediate generation and the site of damage. Although the TrxR-mediated MTZ activation is not designated as the ‘principal’ pathway, remarkably, this enzymatic reaction occurs in the cytosol, where MTZ freely diffuses, making it readily accessible for TrxR, and TrxR-generated reactive intermediates may be responsible for the major protein damage in T. vaginalis. Because TrxR normally transfers electrons from NADPH to thioredoxin, diverting these electrons to nitroimidazole reduction compromises the thioredoxin-peroxiredoxin antioxidant pathway, increasing oxidative stress and thereby opening another avenue to parasite killing with the same weapon. It has not yet been demonstrated whether TrxR inhibition alone is sufficient to confer MTZ resistance in *T. vaginalis*, and there are no quantitative data available to enable comparison of the rates of MTZ reduction by TrxR versus PFOR/ferredoxin under physiological conditions.

This is supported by the clinical MTZ-resistant isolate B7268, which retains functional hydrogenosomal enzymes/factors even under anaerobic conditions, suggesting an alternative extra-hydrogenosomal MTZ activation pathway (Dunne et al., 2003). Amongst differentially expressed genes during early MTZ exposure, classical MTZ activators such as PFOR and iron-sulfur-binding proteins were downregulated, while putative alternative activators, including thioredoxin reductase, nitroreductases, and flavodoxin-like proteins, were upregulated, indicating differential regulation of MTZ activation pathways. MTZ treatment also increased *s*ingle *n*ucleotide *p*olymorphisms (SNPs) and indels, suggesting that MTZ induces DNA damage and genomic alterations in addition to transcriptional changes (Xie et al., 2023).

### 4.2 G. lamblia

Although PFOR remains an important activator of MTZ, the occurrence of high-level MTZ resistance with normal PFOR and Fd activity suggested that the PFOR-mediated activation is not the only pathway in *Giardia*. Using purified enzymes, Leitsch et al. (2011) demonstrated that PFOR and TrxR independently reduce and activate 5-nitroimidazole drugs. TrxR reduces MTZ, oxidizing FAD in the presence of NADPH (**Figure 2B**; step 4 and 5). MTZ-resistant *Giardia* lines exhibited decreased intracellular levels of flavin cofactors (FMN and FAD), thereby compromising TrxR activity. The authors proposed that diminished flavin availability decreases nitroimidazole activation and contributes to resistance. This study also confirmed that ferredoxin I (FdI) efficiently accepts electrons from PFOR and transfers them to MTZ, whereas Fd II and III showed little to no activity in drug activation, highlighting FdI’s preference as the physiological electron acceptor in the classical pathway (Leitsch et al., 2011). Leitsch et al. (2016) provided direct *in vivo* evidence that recombinant *Giardia* TrxR can reduce and activate MTZ, yet pharmacological inhibition of TrxR did not produce a strong resistant phenotype, influencing the authors to conclude that TrxR is a relatively poor candidate for selective chemotherapy.

### 4.3 E. histolytica

TrxR was also identified as a central enzyme in MTZ activation in *E. histolytica*. Leitsch et al. (2007) demonstrated for the first time that *E. histolytica* thioredoxin reductase directly reduces MTZ and other nitro compounds, proposing an alternative activation pathway beyond the classical PFOR-ferredoxin model. Proteomic analysis revealed that MTZ selectively forms covalent adducts with a limited number of cellular proteins. Only five major protein targets were identified by proteomic analysis: thioredoxin (Trx), thioredoxin reductase, superoxide dismutase, purine nucleoside phosphorylase, and one previously uncharacterized protein. Further, Recombinant TrxR carrying MTZ-derived adducts exhibited significantly reduced thioredoxin reductase activity. Subsequently, using an active-site mutant of thioredoxin as an affinity probe, 16 putative thioredoxin-interacting proteins, including peroxiredoxin and enzymes involved in antioxidant defence, metabolism, and protein folding, were identified (Schlosser et al., 2013). The authors suggested that the Trx/TrxR system protects cysteine biosynthesis during oxidative stress. Based on previous findings that MTZ covalently modifies and inactivates both thioredoxin and thioredoxin reductase, their study showed that impairment of this system compromises the function of downstream redox-regulated proteins rather than affecting only the primary drug targets. Inhibition of TrxR by MTZ weakens the parasite’s antioxidant defences and increases susceptibility to oxidative damage. By linking MTZ-mediated inhibition of thioredoxin reductase to downstream disruption of antioxidant defence and cysteine biosynthesis, the authors proposed that the Trx/TrxR network is an attractive target for developing novel anti-amoebic therapies beyond nitroimidazole drugs (Schlosser et al., 2013).

## 5 Mechanistic insights into the nitroreductase-mediated activation of metronidazole

The reduction of nitro groups in nitroimidazole compounds like MTZ is primarily catalysed by the nitroreductase group of enzymes, converting them into reactive cytotoxic intermediates that damage DNA, proteins, and other cellular components. Based on their reaction mechanisms, nitroreductases can either be oxygen insensitive (type I), which catalyse a sequential two-electron reduction of the nitro group, producing stable reduced intermediates or oxygen sensitive (type II), which catalyse a single electron transfer to the nitro group to form a nitro anion radical. The former group members generate stable DNA-damaging intermediates like hydroxylamine and function even under low-oxygen or in microaerophilic conditions (e.g. *E. coli* NfsA and NfsB). On the other hand, oxygen-sensitive nitroreductases of higher eukaryotes are functional only in anaerobic conditions, where in the presence of oxygen, the intermediate compounds rapidly convert back to the original compounds, thus cycling through a futile cycle.

### 5.1 T. vaginalis

Even though laboratory-generated MTZ-resistant *Trichomonas* strains frequently exhibited downregulated expression of hydrogenosome-associated enzymes or factors, such as PFOR and ferredoxin, and sometimes carried an entire deformed hydrogenosome, these observations did not correlate well with those in clinical isolates, which possess normal expression of PFOR and ferredoxin. Clinically isolated *T. vaginalis* may fall into either of the two genetically distinct populations, one of which is more resistant than the other (population 2 and population 1 are 64% and 33% resistant, respectively).

Paulish-Miller et al. (2014) identified SNPs in the nitroreductase genes *ntr4Tv* and *ntr6Tv* that were significantly associated with metronidazole resistance in clinical isolates. These included both nonsense mutations (producing truncated, non-functional proteins) and missense mutations (producing amino acid substitutions with potentially altered function). Additionally, very recently, 304 differentially expressed genes were identified by comparing four MTZ-resistant and four MTZ-sensitive isolates, and among them, five *ntr* genes were found to be downregulated in the resistant isolates, which may be considered as correlative evidence of MTZ reduction and activation by NTR (Graves et al., 2023). However, it is worth mentioning that their findings are essentially based on mRNA expression profile, without proteomic or direct enzymatic experimental validation, which may not reflect the actual scenario at the protein level, for instance, the eventual expression profile of NTRs, their functional efficacy, etc. Although Graves et al. (2023) classified the NTRs as MTZ-detoxifying enzymes, practically, they advocated in favour of the MTZ-activating role of the NTR, as did Paulish-Miller et al. (2014), by SNP and its associated resistance phenotype analyses. Also, NTR enzyme activity was not measured in these mutant strains for comparison with the wild-type strains, and therefore, it is not possible to interpret whether these SNPs were responsible for the loss of its enzymatic activity, leading to reduced MTZ activation. The enzymes may possess context-dependent dual functions, may be linked markers with co-inherited with other resistant determinants, or indirectly associated with metronidazole susceptibility. It is suggested that Ntr8Tv can reduce Fe³^+^ to Fe²^+^, and thereby participate in intracellular iron homeostasis (Paunkov et al., 2025), providing direct biochemical evidence for ferric reductase activity and potentially linking nitroreductase function to the emerging iron-dependent model of MTZ activation and resistance in *T. vaginalis*. The recently proposed cysteine/iron/FR1 pathway requires Fe²^+^ for metronidazole activation (Mayr et al., 2024), loss of nitroreductase-mediated iron reduction could deplete the Fe²^+^ pool, indirectly reducing drug activation and contributing to resistance. However, Direct reduction of MTZ by Ntr8Tv has not been experimentally verified. We suggest verifying whether all NTRs are MTZ-activating or whether a few detoxify the drug by producing aminoimidazole, as in NR2 in *Giardia*.

### 5.2 G. lamblia

In the laboratory-grown MTZ-resistant Giardia strains, the resistance was associated with altered expression of nitroreductases rather than PFOR. MTZ-resistant clones exhibited downregulation of nitroreductase-1, an enzyme involved in drug activation, and upregulation of nitroreductase-2. In contrast, PFOR expression remained largely unchanged, indicating that reduced PFOR activity is not the primary determinant of resistance (Müller et al., 2007a). Two nitroreductases namely GlNR1 and GlNR2 were identified in *Giardia* out of which the GlNR1 did not reduce MTZ, rather it was implicated to bind to the drug and act as a drug-binding buffer, with its inhibition contributing indirectly to redox imbalance (Müller et al., 2007a). This study provides indirect evidence of its buffering role but no direct biochemical evidence of MTZ reduction, leaving the role of GlNR1 inconclusive. This claim was partially annulled by Nillius et al. (2011), who observed that overexpression of GlNR1 increased MTZ susceptibility in *Giardia* trophozoites, supporting its role in drug activation. This observation was explained by the presence of a ferredoxin domain in GlNR1, suggesting that GlNR1 can act as an alternative electron donor in the classical pathway, even in the absence of PFOR. This study provides genetic and functional evidence consistent with activation, but proof of a direct reduction remains unattained. The presence of a ferredoxin domain in GlNR1 can be considered a structural inference suggesting its electron-transfer capability. Therefore, the precise biochemical function of GlNR1 in MTZ metabolism remains unresolved. Structural features suggest electron-transfer capability; overexpression confers susceptibility, but direct reduction of MTZ by purified GlNR1 has not been conclusively demonstrated. Besides, overexpression of GlNR2 in *G. lamblia* trophozoites reduced susceptibility to MTZ. Similarly, when GlNR2 was heterologously overexpressed in nitroreductase-deficient *E. coli* reporter strain SOS-R1, the enzyme did not confer susceptibility to the drug, unlike GlNR1. Consistent with these experimental observations, GlNR2 was either nonresponsive or upregulated in MTZ-resistant strains compared to the susceptible strain (Müller et al., 2013). Therefore, two nitroreductases of *G. lamblia* have distinct roles. GlNR1 is primarily involved in MTZ activation via partial reduction, producing toxic intermediates, whereas GlNR2 appears to mediate complete reduction, yielding non-toxic products and likely inactivating the drug (**Figure 2B**). Heterologous overexpression of GlNR1 in *E. coli* conferred increased drug susceptibility, whereas GlNR2 reduced sensitivity to MTZ, suggesting opposing roles and prompting the authors to propose that GlNR2 has a MTZ-detoxifying rather than activating function (Müller et al., 2015).

Altogether, PFOR, nitroreductase 1, and thioredoxin reductase, all three key enzymes have been linked to the mechanism behind MTZ activation in *G. lamblia*. Unlike *Trichomonas*, *Giardia* shows no spatial separation between these systems, so neither pathway can be identified as preferred for drug activation (Leitsch et al., 2011).

The cumulative effects of MTZ activation include DNA damage and repair mechanisms, inhibition of protein synthesis, oxidative and nitrosative stress, erroneous lipid metabolism and membrane alterations. DNA fragmentation and extensive H2A phosphorylation under MTZ treatment near to its IC50 value for a period of about 12 hours and more, was recorded which may indicate the initiation of DNA repair mechanism in *Giardia* (Uzlikova and Nohynkova, 2014). This treatment also slowed down the progression through the S phase of the cell cycle, likely to allow sufficient time for DNA repair and pass on an unaltered genetic message to the progeny cells (Uzlikova and Nohynkova, 2014). Later, Ordoñez-Quiroz et al. (2018) reported that phosphorylation of H2A triggers the homologous recombination repair pathway as a DNA damage response, as MTZ incorporates double-strand breaks in DNA.

### 5.3 E. histolytica

Nitroreductases, as in *Giardia*, were also identified in *Entamoeba*. Precisely, three copies of oxygen-insensitive (Ehntr1-3) and one copy of oxygen-sensitive (Fdntr) nitroreductases have been described in *E. histolytica* to date. Heterologously overexpressed Ehntr1 and Ehntr2 in the JM109 strain of *E. coli* boost the MTZ sensitivity of this *E. coli* strain (Pal et al., 2009). This group also identified a *Bacteroids fragilis*-like nitroimidazole reductase (NIM), overexpression of which increased MTZ resistance by about 30% in *E. coli* JM109. Additionally, thioredoxin reductase, an important component of redox homeostasis in *Entamoeba*, localised in the underlying plasma membrane (Arias et al., 2008), exhibited *in vitro* nitroreductase activity and is therefore capable of MTZ reduction and activation (**Figure 2C**). Interestingly, binding to MTZ reduces the enzyme’s catalytic activity and lowers intracellular thiol levels due to covalent adduct formation, which was rescued by adding cysteine to the medium. Besides, toxic MTZ intermediates were found to form adducts with *S*uper*o*xide *d*ismutase (SOD), purine nucleoside phosphorylase, thioredoxin, and thioredoxin reductase, thus explaining compromised activity of thioredoxin reductase (Leitsch et al., 2007b). This study provides the first direct evidence of such adduct formation in a living parasite rather than only *in vitro* systems and suggests that this is the major contributing factor to MTZ toxicity **Figure 2C**.

## 6 Cysteine-ferredoxin mediated reduction and activation of metronidazole in *T. vaginalis*

In a very recent study, Mayr et al. (2024) performed a proteomic analysis with two pairs of laboratory sensitive and resistant strains, and two pairs of clinical sensitive and resistant strains of *T. vaginalis*, and found out that Flavin reductase 1 (FR1), an oxygen scavenging enzyme is the only protein that is consistent to be non-functional/downregulated in all resistant cells across all clinical and resistant strains and thus proposed another FR1 mediated MTZ activation pathway. According to this proposal, ferrous iron (Fe²^+^) binds to cysteine residues (free or protein-bound) to form a Fe²^+^[cysteine]_2_ complex, which can either be oxidized by oxygen to ferric iron (Fe³^+^) with concomitant generation of superoxide radicals, or interact with MTZ to form a ternary complex. This complex subsequently reacts with cysteine to generate MTZ nitro radical anions and cysteine thiyl radicals, which damage iron-associated proteins; this process is facilitated by FR1-mediated reduction of Fe³^+^ to Fe²^+^, thereby enhancing drug susceptibility in *Trichomonas vaginalis* (**Figure 3**). According to this model, the ferric-reducing activity of FR1 is a plausible mechanistic bridge between iron homeostasis, Fe²^+^[cysteine]_2_ complex formation, and MTZ reduction; but this pathway is still a hypothesis, and yet to be demonstrated experimentally. Therefore, its role in MTZ activation remains inferential.

**Figure 3:**
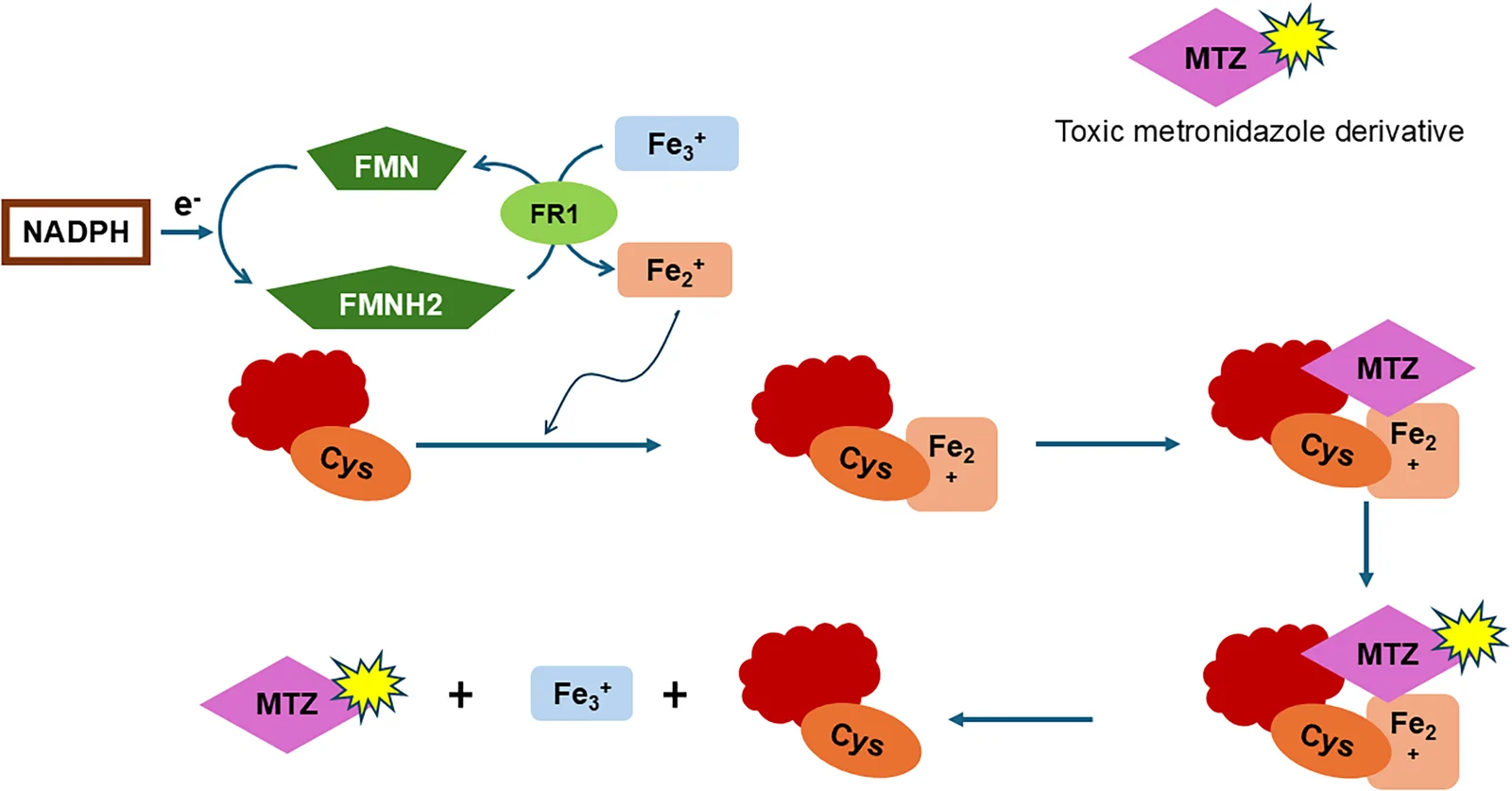
Cysteine-ferredoxin-mediated activation of metronidazole in *T. vaginalis.* Flavin reductase (FR1) accepts electrons from NADPH to reduce FMN, which then transfers electrons to ferric iron (Fe³^+^), generating ferrous iron (Fe²^+^). The Fe²^+^ binds cysteine-containing molecules to form an iron/cysteine complex, which interacts with metronidazole (MTZ) and generates reactive nitro and cysteine-thiyl radicals that can damage proteins.

## 7 Molecular biology behind the mechanism of action of metronidazole in *H. pylori*

*Helicobacter pylori* is a microaerophilic gram-negative bacterium that inhabits the human gastric mucosa causing the disease peptic ulcer. Long-term, untreated infection increases the risk of gastric cancer in the host (Malfertheiner et al., 1997). Functional PFOR is present in *H. pylori*, and the laboratory-derived MTZ-resistants exhibited substantially lower PFOR activity than MTZ-sensitive strains, suggesting a direct relationship between PFOR activity and MTZ susceptibility (Hoffman et al., 1996). Similar to anaerobic protozoans, PFOR of the *H. pylori* was reported to contribute to the MTZ activation. Direct biochemical evidence demonstrates that electrons generated in the catalysis of the pyruvate oxidation are transferred to flavodoxin and further to MTZ under anaerobic conditions (Kaihovaara et al., 1998; van der Wouden et al., 2001), which confirms the previous correlative evidence of compromised PFOR activity in resistant strains, but no evidence has been put forward so far that may advocate for PFOR to be the primary activator in *H. pylori.* It has yet to be established experimentally that, while PFOR can contribute to MTZ reduction in *H. pylori* under certain conditions, RdxA nitroreductase is the primary and rate-limiting activator; PFOR’s role is secondary, or vice versa.

*H. pylori* also possesses multiple nitroreductase genes in its genome, and one such gene is *rdxA* that encodes a classical bacterial-like oxygen-insensitive NADPH nitroreductase but differs in having a lesser cysteine content and a lower isoelectric point (5.4-5.6) than bacteria, which, according to the authors, might explain the nitroreductase’s ability to reduce low redox drugs like MTZ. Loss of RdxA nitroreductase activity results in MTZ resistance and a concomitant 50% reduced PFOR activity in *H. pylori* (Goodwin et al., 1998). The synthesis of truncated/non-functional oxygen-insensitive nitroreductase RdxA due to the nonsense mutations in the *rdxA* gene in MTZ-resistant *H. pylori* clinical isolates implicates the nitroreductase’s reducing property with MTZ reduction and thus activation (Kwon et al., 2000). An isocitrate lyase activity was detected in these resistant strains, which has none or a negligible basal expression in the susceptible strains, suggesting metabolic rewiring towards an alternative pathway. Apart from RdxA, the reduction of MTZ depends on an ‘oxygen scavenging hypothesis’ where higher NADH oxidase activity in sensitive strains reduces oxygen and promotes drug activation. Additionally, the identification of a protein group (Band A), prominent in sensitive strains, and with both oxidase and nitroreductase activities likely participates in redox cycling and contributes reducing power, possibly through interaction with thioredoxin reductase. A major component of this group was identified as AhpC, a homolog of alkyl hydroperoxide reductase (Trend et al., 2001).

FrxA is another oxygen-insensitive NAD(P)H nitroreductase that is involved in MTZ reduction in *H. pylori.* Interestingly, disruption or reduced expression of ferredoxin A (*fdxA*) results in increased *frxA* expression, indicating that ferredoxin participates in a regulatory network in nitroreductase expression rather than merely serving as an electron carrier. Although FrxA contributes significantly to MTZ activation, the persistence of MTZ sensitivity in *H. pylori* strains lacking a functional FrxA suggests its reliance on additional pathways (Mukhopadhyay et al., 2003).

Addition of MTZ directly and competitively inhibits the activity of three disulphide reductase enzymes which are parts of cellular redox homeostasis and use DTNB or Ellman’s reagent, oxidized glutathione, or L-cysteine/ferredoxin as substrates. Competitive inhibition with these substrates indicates the likelihood that MTZ shares the same electron-transfer pathway for its reduction (Kaakoush and Mendz, 2005).

## 8 Factors contributing to metronidazole resistance

Despite its long-standing clinical success, the emergence of MTZ resistance and reduced susceptibility has become an increasing concern, particularly in laboratory-selected strains and, in some cases, clinical isolates. Furthermore, MTZ is less effective in eliminating asymptomatic cyst carriers and is associated with several adverse effects, including gastrointestinal disturbances, dizziness, headache, insomnia, drowsiness, and hypersensitivity reactions. Importantly, *E. histolytica* trophozoites have been shown to survive prolonged exposure to sub-therapeutic concentrations of MTZ and can acquire resistance under *in vitro* conditions (Jeelani and Nozaki, 2019), emphasizing the need to better understand the molecular mechanisms underlying drug activation and resistance to facilitate the development of more effective therapeutic strategies. Here, we congregate the resistance mechanisms against MTZ in the organisms included in this study.

### 8.1 Resistance in *Trichomonas*

In laboratory-selected resistant strains (e.g., RU382), downregulation of ferredoxin (mRNA; 40-65%) was observed (Quon et al., 1992). However, this anaerobic resistance is distinct from the aerobic resistance that is commonly observed in clinical isolates, which is primarily associated with defective oxygen-scavenging (FR1 deficiency) ability rather than with hydrogenosomal enzyme downregulation. A G>A mutation at 178 bp upstream and an A>T mutation at 239 bp upstream of the ferredoxin coding sequence were identified in the resistant strain RU382 (Quon et al., 1992). Transformation of *E. coli* JM109 with *Trichomonas* Nitroimidazole reductase (NIM) genes resulted in MTZ resistance in the transformed cells (Pal et al., 2009). Nonsense mutation in *Tvnim1* and/or truncated *Tvnim3* gene was detected in some *Trichomonas* strains. Clinical isolates of *T. vaginalis* may develop aerobic resistance in treatment-refractory patients. Therefore, two distinct resistance phenotypes have been described in *T. vaginalis*: (1) Aerobic or clinical resistance, observed in treatment-refractory clinical isolates, characterized by impaired oxygen-scavenging (FR1 deficiency) and elevated intracellular oxygen; and (2) Anaerobic (laboratory-selected) resistance, generated through *in vitro* selection, characterized by the loss of hydrogenosomal enzymes (PFOR, hydrogenase, ferredoxin, malic enzyme). The relative contribution of anaerobic resistance mechanisms to clinical treatment failure remains unclear. This supports the concept of an alternative MTZ activation pathway (Rasoloson et al., 2002). Under aerobic condition, the MTZ in resistant clinical isolate BRIS/92/STDL/B7268 (B7268) has an MIC (*m*inimum *i*nhibitory *c*oncentration) > 200 µM, whereas, in anaerobic condition, it has an MIC of 25 µM, while the sensitive isolates typically show no growth at an MIC of 3-6 µM (Dunne et al., 2003). Here, the authors indicate that the downregulation of hydrogenosomal metabolism, including decreased hydrogenase function, reduces intracellular MTZ activation and contributes to drug resistance, but emphasize that hydrogenase is only one component of a broader resistance mechanism involving coordinated changes in PFOR, ferredoxin, oxygen metabolism, and other hydrogenosomal redox pathways, with clinical resistance often differing from the classical laboratory-induced hydrogenosomal suppression model (Dunne et al., 2003). The B7268 strain also lacks a detectable amount of *F*lavin *r*eductase 1 (FR1) which is the only isoform of FR with an affinity towards flavins out of seven full-length and three truncated isoforms, and provides antioxidative protection to *Trichomonas* by reducing molecular oxygen to H_2_O_2_. Without a functional FR1, molecular O_2_ is not reduced, rather competes with MTZ for electrons resulting in the futile cycling of intermediate MTZ derivatives to produce MTZ back again (Leitsch et al., 2014). Complementation of B7268 with a functional FR1 extrachromosomally could almost fully restore its sensitivity to MTZ. This supports a previous observation of increased oxygen susceptibility in MTZ-resistant strains (Rasoloson et al., 2001). However, unlike *T. vaginalis* FR1, *T. foetus* FR was not found to be associated with MTZ resistance (Gehl et al., 2021).

Out of eleven previously identified *ntr* genes, *ntr4_Tv_*, *ntr6_Tv_*, *ntr9_Tv_*, and *ntr10_Tv_*carried several SNPs, and amongst them, SNPs present in only *ntr4_Tv_* and *ntr6_Tv_* were associated with MTZ resistance. In particular, *ntr4Tv* C213G (Y71STOP) and *ntr6Tv* A238T (K80STOP) nonsense mutations were observed to have the strongest association with the resistance phenotype (Paulish-Miller et al., 2014). Interestingly, these nonsense mutations were more enriched in population 2 than in population 1; for example, the frequencies of *ntr4Tv* Y71STOP in populations 1 and 2 were 4.7% and 88%, respectively, whereas the frequencies of *ntr6Tv* K80STOP in these two populations were 0% and 38%, respectively.

Apart from these two nonsense mutations, which produce truncated and likely non-functional NTRs, a few missense mutations were also detected with substantial significance. These are D26H and H106Q in *ntr4Tv*, and E143Q and V159A in *ntr6Tv*, which may alter protein stability, enzymatic activity, and other functions, but may not culminate in the synthesis of NTR without enzymatic activity. These variants may have very different relationships with the resistant phenotype; for example, *ntr6Tv* E143Q was found in 76% of resistant isolates and 36% of susceptible isolates, whereas K80STOP occurred in 32% of resistant isolates and 4.1% of susceptible isolates, for which the authors propose these SNP as a disease marker. These missense variants function as multivariant haplotypes, since E143Q was found alone as well as with K80STOP and V159A in 22 out of 100 isolates. Similarly, D26H and Y71STOP in *ntr4Tv* always occurred together. However, the functional interpretation of these mutations requires careful consideration. Establishing the association of SNPs with resistance doesn’t determine the causal direction until experimentally validated; therefore, in the absence of experimental data on enzymatic activities, it is difficult to assign their role in the development of MTZ resistance. Several sequencing, transcriptome, and proteome analyses have been conducted to identify genetic markers, novel proteins associated with MTZ resistance, or molecular targets of MTZ in *Trichomonas*. By sequencing a number of MTZ-resistant clinical isolates and lab-derived lines, Bradic et al. (2017) found 72 SNPs associated with the resistance. Transcriptome analysis revealed downregulation of MTZ activation-associated genes, upregulation of detoxification-associated nitroreductase genes as well as overexpression of an ABC transporter (TVAG_254060) and two multidrug resistance pumps (TVAG_291970 and TVAG_210540). Out of all nitroreductases, nitroreductase 8 could reduce ferric ion, indicating that it has the potential to reduce MTZ as well. Therefore, its downregulation may contribute to MTZ resistance. However, the efflux pumps were not found to be involved in the MTZ resistance (Paunkov et al., 2025). A comparison of proteomic data from iTRAQ analyses identified 228 upregulated and 76 downregulated genes in the MTZ-resistant strain compared to the sensitive strain. This comparison, followed by a STRING and gene set enrichment analysis (GESA) suggested that the amino acid metabolism pathway components had an upregulation in expression, while the factors associated with the oxidative phosphorylation were downregulated in the resistant strain, of which ten proteins fall into the ATP synthase subunit family proteins, indicating a potential contribution to the drug susceptibility (Lin et al., 2020). Similar results were obtained by Huang et al. (2021), along with a few previously unidentified genes like 5′-nucleotidase surE and Na^+^-driven multidrug efflux pumps. Additionally, diminished purine and pyrimidine metabolism, but, hyperactive *E*ndoplasmic *R*eticulum-*A*ssociated *D*egradation (ERAD) pathways are some of the striking findings obtained by functional enrichments. Graves et al. (2023) also observed an upregulation in the multidrug resistance pump and metal *ABC transporter* genes were upregulated in all three resistant strains. On the other hand, Mayr et al. (2024) pointed out by performing a proteomic analysis, that FR1 is the only protein that is consistent to be non-functional in all resistant cells across all independent experiments (**Table 2**). Both laboratory-derived and clinical isolates of resistant strains provide a strong foundation for understanding FR1’s contribution to MTZ resistance in *T. vaginalis* (Mayr et al., 2024).

**Table 2:** Mechanism of metronidazole resistance in *Trichomonas*.

Resistance mechanism	Key factors involved	Effect on metronidazole activation/susceptibility
Deficient oxygen scavenging (aerobic/clinical resistance)	Flavin reductase 1 (FR1), NADH oxidase, antioxidant defense pathways	Downregulation or impairment of oxygen-scavenging pathways increases intracellular oxygen. Oxygen competes for electrons and re-oxidizes reduced metronidazole intermediates (futile cycling), thereby limiting formation of toxic nitro radicals and reducing drug activity (Leitsch, 2016; Graves et al., 2020).
Impaired hydrogenosomal activation (anaerobic resistance)	Pyruvate:ferredoxin oxidoreductase (PFOR), ferredoxin (Fd), hydrogenase	Reduced expression or loss of hydrogenosomal enzymes decreases metronidazole reduction within the hydrogenosome, thereby limiting production of toxic radicals (Rasoloson et al., 2002; Graves et al., 2020).
Nitroreductase-associated resistance	*ntr4Tv*, *ntr6Tv* and related nitroreductases	Mutations or altered expression reduce the ability of nitroreductases to convert metronidazole into toxic radical intermediates, thereby decreasing susceptibility (Ozcelik et al., 2018; Ngobese et al., 2022)
Global proteomic and metabolic adaptation	Amino acid metabolism proteins, oxidative phosphorylation components, ATP synthase-associated proteins, stress-response proteins	Broad metabolic remodeling alters intracellular redox balance and indirectly affects drug activation and tolerance (Lin et al., 2020).
Aerobic (clinical) resistance phenotype	Primarily defective oxygen-scavenging systems	Elevated intracellular oxygen suppresses nitro radical formation and decreases metronidazole toxicity (Graves et al., 2020).
Anaerobic (laboratory-selected) resistance phenotype	Loss/downregulation of hydrogenosomal drug-activating enzymes	Markedly impaired intracellular activation of metronidazole under anaerobic conditions (Rasoloson et al., 2002).
Genetic polymorphism-mediated resistance	Mutations in nitroreductases and other metronidazole-associated genes	Genetic alterations may predispose isolates to reduced susceptibility by lowering activation efficiency (Ngobese et al., 2022).

### 8.2 Resistance in *Giardia*

MTZ resistance in *Giardia* is multifactorial and not due to a single mechanism. Resistance arises from a combination of reduced drug activation, metabolic adaptations, altered redox systems, and genetic/transcriptional changes (**Table 3**). The diminished activity of enzymes involved in the reduction and activation of MTZ, such as nitroductase-1, thioredoxin reductase, and pyruvate-ferredoxin oxidoreductase (PFOR), has been linked to mechanisms underlying MTZ resistance in *G. lamblia*. Most of the laboratory-selected MTZ-resistant *Giardia* strains exhibited at least 5-fold reduction in PFOR activity (Townson et al., 1996). However, the prevalence of PFOR downregulation in clinically resistant isolates has not been systematically evaluated till today. Impaired NADPH oxidase activity disrupts thiordoxin-mediated flavin metabolism and MTZ reduction, thus contributing to resistance development too (**Figure 2B**; step 5) (Leitsch et al., 2011). Resistance can also develop through detoxification of toxic radicals, and drug efflux mechanisms, rather than a single pathway alteration. The finding of Müller et al. (2007a) was based on laboratory-selected resistant clones rather than clinical isolates. In laboratory-selected resistant *Giardia* lines, reduced GlNR1 and increased GlNR2 expression were observed; the clinical relevance of these changes remains to be established. Cross-resistance to MTZ and albendazole (Upcroft, 1998) was documented, supporting the theory of multidrug resistance by mammalian *mdr* homolog-associated labelled MTZ uptake, transport, and efflux from cytoplasm (Upcroft, 1994) which was previously described as low uptake of MTZ by resistant *Giardia* due to a compromised diffusion of the drug through plasma membrane (Boreham et al., 1988; Townson et al., 1996). An upregulation in *P*rotein *D*isulphide *I*somerase (PDI) 2 and 4 was observed in the resistant cells, likely to compensate for the drug-induced inhibition of protein folding enzymes. Additionally, significant down-regulation of *V*arious *S*urface *P*roteins (VSPs; surface antigens), especially TSA417 may indicate a broader adaptation and plausible epigenetic changes contributing to drug resistance (Müller et al., 2007a).

**Table 3:** Mechanism of metronidazole resistance in *Giardia*.

Resistance mechanism	Key factors involved	Effect on metronidazole activation/susceptibility
Reduced drug activation by electron transfer enzymes	Pyruvate:ferredoxin oxidoreductase (PFOR) and ferredoxin	Reduced supply of low-redox electrons decreases intracellular activation of metronidazole and limits formation of toxic radical intermediates (Leitsch, 2015).
Altered nitroreductase balance	Nitroreductase-1 (NR1) and nitroreductase-2 (NR2)	Downregulation of NR1 reduces activation of metronidazole, whereas upregulation of NR2 promotes complete reduction of the nitro group to a non-toxic amino derivative, thereby enhancing detoxification. toxicity (Leitsch, 2015).
Altered flavin metabolism and redox enzyme activity	Thioredoxin reductase and other flavin-dependent oxidoreductases	Reduced flavin-mediated electron transfer limits metronidazole reduction and contributes to resistance (Leitsch et al., 2011).
Passive resistance through reduced activation capacity	Reduced activity of activation enzymes such as PFOR and NR1	Lower intracellular concentrations of activated metronidazole radicals enhance trophozoite survival. (Ansell et al., 2015).
Global transcriptional adaptation	Differential expression of PFOR, NR1, NR2, stress-response genes, antioxidant systems, and variant surface proteins (VSPs)	Coordinated transcriptional changes simultaneously reduce drug activation and increase detoxification/stress tolerance. (Ansell et al., 2015).
Genetic variation associated with reduced susceptibility	Mutations and variants in PFOR, NR, and other metronidazole-associated genes	Genetic alterations may reduce enzyme functionality and predispose strains to reduced drug susceptibility (Saghaug et al., 2023).

*Giardia* lacks most of the enzymes and molecules required for oxidative stress management, like catalase, SOD, peroxidase, GSH, etc., but under aerobic conditions, the toxic MTZ derivative is reverted back to its origin in the presence of molecular O_2_ with the production of superoxide radical (**Figure 2B**; step 6) (Leitsch et al., 2011). *Giardia* with strong MTZ resistance did not show anti-SOD activity, but 1, 10-phenanthroline can induce Fe-SOD in *Entamoeba* (Townson et al., 1996). Genetic variability in the principal genes of the classical and alternative MTZ activation pathways is often reported in the resistant clinical isolates. Non-synonymous single-nucleotide variations in genes associated with oxidative and nitrosative stress management were revealed by whole-genome sequencing in these isolates (Saghaug et al., 2019).

### 8.3 Resistance in *E. histolytica*

Similar to *Giardia*, MTZ resistance in *E. histolytica* appears to be multifactorial and is best characterised in laboratory-induced resistant lines rather than in common clinical isolates. Changes in the enzyme structure and activity involved in antioxidant defence and electron transfer are demonstrated as probable mechanisms underlying reduced drug susceptibility in *Entamoeba* (**Table 4**). Although *E. histolytica* is primarily an anaerobic protozoon, in the lumen of the gastrointestinal tract of the host system, it is exposed to an aerobic environment, and this microaerophilic condition gives rise to resistant strains, which directly correlate with a significant increase in Fe-SOD expression (Samuelson, 1999; Wassmann et al., 1999). Fe-SOD is central to detoxifying the toxic effect superoxide radical generated by host immune cells and its own metabolism and converting the radical to hydrogen peroxide (**Figure 4**). Peroxiredoxin and Flavin reductase enzymes have H_2_O_2_-removing ability. Although peroxiredoxin upregulation alone was not sufficient to confer MTZ resistance, in laboratory-selected MTZ-resistant *E. histolytica*, the combined overexpression of Fe-SOD and peroxiredoxin conferred reduced MTZ sensitivity (Wassmann et al., 1999). Whether such overexpressions occur in clinically resistant isolates as well remains to be determined. Additionally, downregulation of Ferredoxin 1, but not Ferredoxin 2, and compromised activity of Flavin reductase were also implicated in resistance development in *E. histolytica* (Wassmann et al., 1999). Although its prevalence in a large clinical cohort has yet to be established, the nonsense mutation in *Ehntr1* is one of the few identified mutations associated with MTZ resistance in clinical *E. histolytica* isolates, and therefore carries an immense significance in medical parasitology. EhNTR1, carrying this nonsense mutation, introduces an intron to the CDS, causing the subsequent removal of amino acids from the nitroreductase 1. A similar nonsense mutation was found in the *ntr* gene of MTZ-resistant *H*. *pylori* (Pal et al., 2009). It is worth noting that NIM inactivates MTZ by reducing its nitro group completely to a non-toxic amino derivative, aminoimidazole (**Figure 2C**). However, the expression of NIM does not change with the susceptibility of *Entamoeba* to MTZ, and therefore, MTZ resistance could not be directly attributed to NIM activity (Pal et al., 2009).

**Table 4:** Mechanism of metronidazole resistance in *Entamoeba*.

Resistance mechanism	Key factors involved	Effect on metronidazole activation/susceptibility
Enhanced oxidative stress defence	Iron-containing superoxide dismutase (Fe-SOD), peroxiredoxin (Prx)	Increased detoxification of reactive intermediates lowers tde cytotoxic effects of activated metronidazole (Samarawickrema et al., 1997; Wassmann et al., 1999; Upcroft and Upcroft, 2001).
Altered ferredoxin-dependent activation	Ferredoxin (Fd)	Reduced electron transfer decreases conversion of metronidazole to toxic nitro-radical intermediates (Wassmann et al., 1999).
PFOR-independent resistance pattern	Pyruvate:ferredoxin oxidoreductase (PFOR)	Unlike in some other anaerobic protozoa, resistance may occur without major PFOR downregulation (Samarawickrema et al., 1997; Penuliar et al., 2015).
Transcriptional remodeling and metabolic adaptation	Iron-sulfur flavoproteins, stress-response genes, hypothetical proteins	Global transcriptional changes may alter intracellular redox balance and reduce effective drug toxicity (Penuliar et al., 2015).
Redox maintenance pathway alterations	NADPH-dependent oxidoreductases, cysteine-linked redox metabolism	Modified intracellular redox homeostasis may limit metronidazole activation or neutralize toxic intermediates (Jeelani et al., 2010).

**Figure 4:**
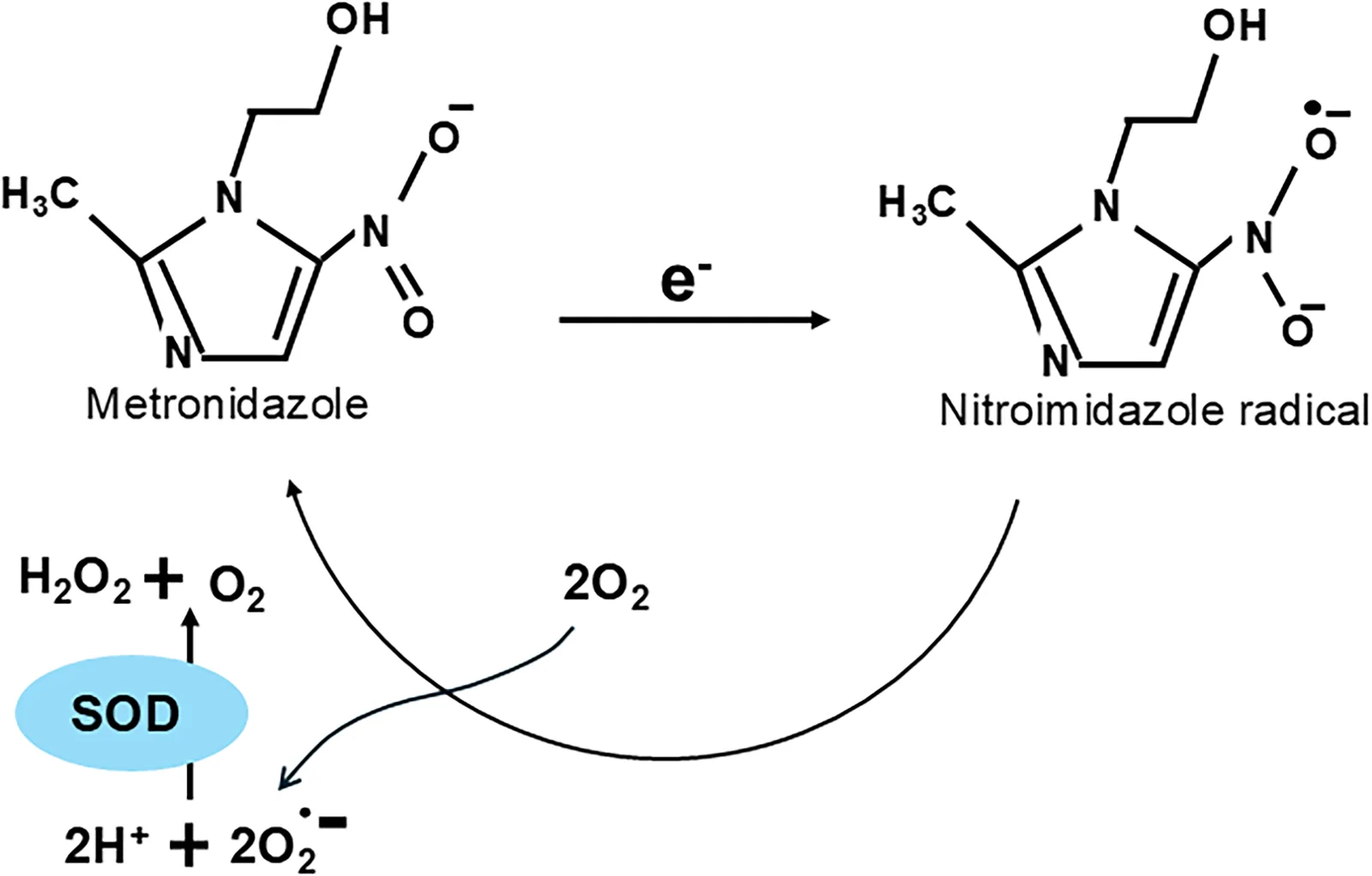
Metronidazole detoxification by Fe-SOD. In *E. histolytica*, under aerobic condition nitroimidazole reductase is converted back to metronidazole through a futile cycle producing free oxygen radical which is scavenged to hydrogen peroxide and oxygen molecule by the superoxide dismutase activity.

### 8.4 Factors contributing to metronidazole resistance *in H. pylori*

In contrast to the protozoan pathogens discussed above, where much of the resistance data derive from laboratory-selected strains, the genetic basis of MTZ resistance, with RdxA inactivation as the predominant mechanism in *H. pylori*, is well established in clinical isolates. Bacterial factors linked to *H. pylori* resistance are mutations in associated genes, efflux pumps, and biofilm formation. Inactivation of nitroreductase RdxA as the primary mechanism of MTZ resistance was first proposed by Kwon et al. (2000). Nonsense mutations in the *rdxA* gene of *H. pylori* lead to the production of truncated, non-functional oxygen-insensitive RdxA (Kwon et al., 2000). This study also demonstrated that *in vitro* inactivation of *frxA* gene, encoding another nitroreductase, was associated with resistance development. Clinically resistant isolates often harboured nonsense mutations in *rdxA*, most of which were indels, along with one substitution mutation introducing a stop codon. Goodwin et al. (1998) supported this with biochemical evidence. Comparative sequence analysis of sensitive and resistant clinical isolates confirmed that only RdxA inactivation due to nonsense or frameshift mutations producing truncated, non-functional protein is sufficient to confer MTZ resistance; FrxA inactivation alone could not confer resistance but displayed an enhanced resistance phenotype in *rdxA*-deficient strains (Kim et al., 2009). Although resistant *H. pylori* cells frequently harbour frameshift mutations, indels, and/or premature stop codons in the *frxA* ORF (Mukhopadhyay et al., 2003), truncated FrxA was found in the 50% of the sensitive clinical strains with functional RdxA, whereas 89% of resistant clinical isolates carried mutations in the *rdxA* gene (Yang et al., 2004). It was later discovered by *in silico* analysis that mutations in the FMN-binding sites weaken drug interaction (Chu et al., 2020). The *fdxB* (a ferredoxin-like protein) is an additional contributing factor to enhanced resistance, particularly when combined with *rdxA* (Kwon et al., 2000).

However, several instances of resistance without *rdxA/frxA* mutations have been recorded, such as broader redox remodelling involving ferredoxin-linked oxidoreductases, TrxR, AhpC, and SOD can downregulate MTZ activation (Kaakoush et al., 2009); RdxA/FrxA independent and oxygen-dependent resistance occurs through the futile cycling of MTZ (Gerrits et al., 2004). Complete loss of TolC-like efflux proteins (HP0605 and HP0971) increased MTZ susceptibility independent of *rdxA* or *frxA* mutations, indicating a role of efflux pumps in resistance development (van Amsterdam et al., 2005). Interestingly, active expulsion of MTZ by two efflux pumps, *hp1165* and *hefA*, both of which are highly expressed in biofilm-forming cells, reduces intracellular MTZ availability, thereby increasing resistance (Attaran et al., 2017).

MTZ is remarkably mutagenic in *H. pylori*, particularly in strains with a functional rdxA gene. MTZ induces DNA damage as already described, and can both select for and promote resistance by generating loss-of-function mutations in *rdxA*. This creates a self-perpetuating resistance cycle: functional RdxA ✉ MTZ activation ✉ DNA damage ✉ mutagenesis in *rdxA* ✉ RdxA inactivation ✉ resistance (Sisson et al., 2000). The strong association between *rdxA* mutations and MTZ resistance may suggest that *rdxA* mutation prevalence may overestimate its relative contribution compared to other mechanisms that are less mutation-prone.

Therefore, it can be interpreted that RdxA inactivation is the most common, strongest, and most frequently observed determinant of MTZ resistance in *H. pylori*, explaining the occurrence of non-functional RdxA in the large majority (approximately 89%) of clinical isolates. However, it is not the only determinant, as a small fraction of resistant strains with functional RdxA may develop resistance attributable to alternative mechanisms.

It is noteworthy that not all *rdxA* mutations confer MTZ resistance. Only loss-of-function mutations (nonsense, frameshift, FMN-binding site disruption) were found to reduce interaction with the drug and contribute to resistance in *in silico* (Chu et al., 2020) and experimental analyses (Solcà et al., 2000; Thomas and Gwenin, 2021). Missense mutations in *rdxA* were found to be non-contributory, indicating that previously reported amino acid substitutions likely represent natural variants rather than resistance determinants. Therefore, sequence-based screening for *rdxA* mutations is not sufficient to predict resistance. The functional impact of each mutation must be assessed. **Table 5** summarizes the MTZ resistance mechanisms in *H. pylori*.

**Table 5:** Mechanism of metronidazole resistance in *Helicobacter*.

Resistance mechanism	Key factors involved	Effect on metronidazole activation/susceptibility
Reduced prodrug activation by nitroreductase inactivation	RdxA (oxygen-insensitive NADPH nitroreductase)	Inactivating mutations reduce conversion of metronidazole into toxic nitro-radical intermediates, thereby lowering intracellular drug activity (Jenks and Edwards, 2002; Kwon et al., 2000).
Additional contribution of flavin oxidoreductase defects	FrxA (NADPH flavin oxidoreductase)	Reduced flavin-dependent reduction further limits metronidazole activation and may enhance resistance, especially when combined with *rdxA* defects. (Kwon et al., 2000).
Oxygen-dependent (redox-mediated) resistance	Intracellular oxygen tension, electron transport/redox metabolism	Oxygen competes for electrons and re-oxidizes partially reduced metronidazole (futile cycling), reducing formation of toxic radicals (Gerrits et al., 2004).
Complex redox remodeling beyond classical nitroreductases	Ferredoxin-linked oxidoreductases, thioredoxin reductase, alkyl hydroperoxide reductase, superoxide dismutase	Broader metabolic shifts alter intracellular redox potential and can reduce metronidazole activation even in the absence of *rdxA*/*frxA* mutations (Kaakoush et al., 2009).
Diverse mutational pathways in clinical isolates	*rdxA*, *frxA*, and their upstream regulatory regions	Multiple genetic alterations can reduce enzyme function or expression, generating heterogeneous resistance phenotypes (Tshibangu-Kabamba and Yamaoka, 2021).

The extent of clinical validation of resistance mechanisms across all organisms discussed in this review is summarized in **Table 6**.

**Table 6:** Mechanisms of metronidazole resistance and the strength of supporting clinical evidence.

Organism	Clinically validated mechanisms	Strength of clinical evidence
*T. vaginalis*	FR1 deficiency (aerobic resistance); Ntr4Tv/Ntr6Tv SNPs	• **Strong** for FR1 (complementation) • **Moderate** for NTR SNPs (association only)
*G. lamblia*	None robustly validated	• **Weak**; most data from laboratory strains
*E. histolytica*	Ehntr1 nonsense mutation (3/22 isolates)	• **Weak to moderate**; limited sample size
*H. pylori*	RdxA inactivation (predominant); FrxA inactivation (secondary)	• **Very strong**; multiple large clinical studies

## 9 Metronidazole-induced cell death in anaerobic protozoans

For multicellular organisms, cell death is an essential and unavoidable process. The targeted elimination of irreversibly damaged, senescent, and infected cells is a prerequisite for maintaining a healthy and effective physiological system and supporting normal development of the organism. The function of mitochondria in the execution of coordinated programmed cell death in higher eukaryotes is well-established, but not all protozoan species possess a true mitochondrion. Similarly, caspases involved in extrinsic or intrinsic apoptotic pathways are not always present in these primitive organisms. However, caspase-dependent, whenever present, or non-classical caspase-independent apoptotic pathways have been reported several times in such parasitic protozoan groups (Kaczanowski et al., 2011). Below, we discuss the cell death pathways reported to date in *T. vaginalis*, *G. lamblia*, and *E. histolytica*, with a particular focus on those relevant to MTZ treatment.

### 9.1 Cell death in *Trichomonas*

In *T. vaginalis*, chromatin condensation and DNA fragmentation exemplify classical apoptotic markers (Chose et al., 2002), yet not sufficient to establish apoptosis, whereas the overexpression of metacaspase (Ebrahimi et al., 2021) are suggestive of caspase-like activity that can be inferred to apoptotic cell death. ERAD-mediated pathway, ubiquitin-mediated proteolysis, and proteasomal system activation (Huang et al., 2021) are stress-response pathways that may precede either apoptosis or necrosis and thus should not be referred to as initiation hallmarks of either. In contrast to the apoptotic morphologies, the recent demonstration of features like membrane rupture, deformed nuclei, and organelle destruction by Xie et al. (2023) typically associates with necrosis or late-stage apoptosis (secondary necrosis). The global transcription shutdown, compromised motility, and impaired signal transduction are also indicative of catastrophic cellular collapse. The co-occurrence of ER stress responses, proteasomal activation, and morphological features of both apoptosis and necrosis suggest that *Trichomonas* may undergo a hybrid, multi-modal death process that cannot be reduced to a single canonical category. Therefore, none of the observed features, alone or in combination, permits definitive classification as apoptosis, necrosis, necroptosis, pyroptosis, or parthanatos according to current NCCD criteria (Galluzzi et al., 2018), thereby necessitating further investigation.

### 9.2 Cell death in *Giardia*

*Giardia* exhibits morphological characters consistent with both programmed and non-apoptotic cell death. Nuclear degradation and chromatin dispersion are the early morphological changes, followed by apoptosis-like programmed cell death, such as membrane blebbing (Ghosh et al., 2009; Bagchi et al., 2012). DNA double-strand breaks and cell-cycle arrest further indicate the activation of a DNA damage response preceding cell death (Uzlikova and Nohynkova, 2014; Ordoñez-Quiroz et al., 2018). These events involve oxidative stress, elevated ROS levels, and lipid peroxidation, which may indicate the commencement of multiple cell death pathways such as ferroptosis and parthanatos (Raj et al., 2024). Together with this, the disruption of glycerophospholipid metabolism, membrane damage, and inhibition of protein synthesis, suggest necrotic or ferroptotic cell death (Popruk et al., 2023). However, the lack of evidence of classical caspase activation argues against canonical apoptosis (Ghosh et al., 2009; Bagchi et al., 2012), which aligns with the apparent lack of classical mitochondria in *Giardia*, making the occurrence of a conventional mitochondria-dependent death pathway unlikely.

The NCCD guidelines (Galluzzi et al., 2018) recognize apoptotic morphology without non-canonical execution as apoptosis-like programmed cell death and apoptotic features but without caspase involvement as caspase-independent apoptosis-like PCD. When metacaspases are the executing proteases, the process is known as metacaspase-mediated regulated cell death.

The execution process of death in *Giardia* may be more catastrophic and less regulated than in organisms with redundant antioxidant defences. Membrane blebbing is generally considered a caspase-dependent process, which makes it intriguing in *Giardia*, where caspases are absent. Therefore, it can be mediated by metacaspases due to ROS-mediated actin oxidation and/or by osmotic or ionic dysregulation. Being deficient in conventional detoxifying enzymes, such as catalase, SOD, peroxidase, and glutathione reductase, this protozoan relies on thioredoxin/peroxiredoxin systems for antioxidant defence. This could explain the simultaneous observation of apoptotic-like features (chromatin dispersion, blebbing) and necrotic features (membrane damage, metabolic collapse). The cell may initiate a regulated death program, but lacking the machinery to execute it neatly, eventually degenerates into a necrotic phenotype. Since iron is essential for *Giardia* survival and is actively acquired from the host, MTZ-induced cell death in *Giardia* may be accomplished through ferroptosis. The combination of oxidative stress, lipid peroxidation, membrane damage, and impaired antioxidant defence (due to TrxR inhibition) is also mechanistically consistent with ferroptosis.

### 9.3 Cell death in *Entamoeba*

Being a primitive protozoan, *Entamoeba* possesses a rudimentary form of mitochondria called ‘mitosome’ or ‘crypton’, rather than fully formed typical mitochondria (Ghosh et al., 2000; Tover et al., 1999). According to several study teams, hydrogen peroxide (H_2_O_2_) can induce apoptotic features such as DNA fragmentation, increased vacuolization, phosphatidylserine externalization, intracellular ROS production, and cell shrinkage in *E. histolytica* (Ghadirian et al., 1986; Ghosh et al., 2010; Nandi et al., 2010). Drugs like resveratrol and G418 also reportedly induce apoptotic morphology (Pais-Morales et al., 2016; Villalba et al., 2007). Similar to *Girdia*, PS externalization in *E. histolytica* under oxidative stress, which is a classical early apoptotic marker of eukaryotes, could be the result of either apoptosis-like death or membrane perturbation. Leitsch et al. (2007) demonstrated that inhibition of the thioredoxin-dependent redox system is a major contributor to parasite killing. Activated MTZ forms covalent adducts with free cysteine and other non-protein thiols, causing a marked depletion of the parasite’s intracellular thiol pool and disrupting redox homeostasis. Supplementation of the culture medium with cysteine significantly improves parasite survival and reduces protein adduct formation, demonstrating that intracellular thiol availability strongly influences MTZ susceptibility. The authors suggest that TrxR both activates MTZ and subsequently becomes one of its primary molecular targets. This creates a self-amplifying cycle in which drug activation simultaneously inactivates the parasite’s major redox defence system, leading to widespread oxidative damage and cell death. MTZ executes apoptosis in *E. histolytica* through an *A*poptosis-*i*nducing *F*actor (AIF)-mediated and caspase-independent pathway (Bandyopadhyay and Ghosh, 2023a). AIF translocate to the nucleus from cytoplasm and carries out DNA fragmentation and chromatin condensation. Additionally, MTZ also increases intracellular ROS levels and initiates nucleophagy, and these synergistic effects lead to cell death (Bandyopadhyay and Ghosh, 2023b). The interaction between AIF and ATG8 suggests a crosslink of two pathways where nuclear AIF promotes phagosome formation around the damaged nucleus (Bandyopadhyay and Ghosh, 2023b). Caspase-independent and AIF-mediated cell death with nuclear translocation of AIF and DNA fragmentation are the characteristic features of parthanatos, a distinct regulated cell death process (Wang et al., 2011; Fatokun et al., 2014). PARP-1 hyperactivation as the upstream trigger of AIF translocation is a prerequisite for parthanatos in mammalian cells. Should PARP-1 not be detected, the AIF-mediated death in *Entamoeba* may represent a parthanatos-like process that is evolutionarily related to, yet distinct from, the mammalian pathway, possibly triggered by oxidative stress or DNA damage.

Autophagic cell death is another NCCD-recognized cell death mode of eukaryotes (Galluzzi et al., 2018), the features of which are partly fulfilled by the primitive protozoan *E. histolytica*. Notably, *Entamoeba* death is associated with redox catastrophe. Therefore, the MTZ-induced cell death mode in *Entamoeba* may be described as an AIF-mediated, caspase-independent regulated cell death with features of parthanatos and autophagic cell death, driven by oxidative stress and redox imbalance.

### 9.4 Cell death in *Helicobacter*

Although programmed cell death, including apoptosis, is generally considered a characteristic feature of multicellular eukaryotes, employment of regulated cell death as an ancient immune response to phage infection in bacterial population slowing down the spread of infection is a remarkable discovery advocating that eukaryotic immune components has evolved from prokaryotic immune system (Rousset and Sorek, 2023). Although *Helicobacter* is well-known for inducing cell death in their host systems (Neu et al., 2002; Shibayama et al., 2003), hardly any study aiming to understand the cell death pathways in *H. pylori* has been carried out so far. However, the manifestation of apoptosis-like morphologies, such as DNA damage and PS ectropionation, by linolenic acid-MTZ complex-induced oxidative stress effectively killed *H. pylori* (Zhou et al., 2023). This observation certainly carries an enormous significance because this phenomenon is typically considered a eukaryotic-specific apoptotic marker. While the oxidative stress and DNA damage may represent features of regulated death, in the absence of other eukaryotic components, the PS externalization may indicate membrane perturbation and accidental death. Should MTZ-induced cell death happen in *H. pylori*, it is strongly likely that it would be a kind of bacterial programmed cell death rather than the NCCD-recognized eukaryotic type of cell death (Bayles, 2014; Allocati et al., 2015; Rousset and Sorek, 2023), and this certainly offers a promising field of further research.

## 10 Future of treatment; alternative approaches

### 10.1 Future of trichomoniasis treatment

It is important to find new drug targets with new mechanistic approaches to combat the rising concerns with nitroimidazole-resistant *Trichomonas* strains. 4’-Hydroxychalcone shows promising result in trophozoite killing by inducing co-cultured neutrophils to generate ROS. This compound is also predicted to be a ligand of purine nucleoside phosphorylase of *T. vaginalis* (TvPNP), a well-known virulence factor, by *in silico* analysis (Oliveira et al., 2023). Induction of global gene transcription by inhibiting histone deacetylases with trichostatin A, alone or in combination with MTZ, induces the expression of silenced/downregulated genes including those involved in the oxidoreductase activity, redox homeostasis etc. in MTZ-resistant *Trichomonas* suggesting that it might be a promising approach to defeat the highly MTZ-resistant *T. vaginalis* cells (Seifert-Gorzycki et al., 2025). A comprehensive outlook on the potent targets for the treatment of *T. vaginalis* infection sheds light on autophagy and proteasomal components like F-dAdo, the substrate of TvPNP, and TVAP33, a well-known virulence factor etc. to be considered as future drug targets (Singh et al., 2025). Repurposing of MTZ following analysis of structure-activity relationships to reveal probable structural modifications that would influence drug activation, cytotoxicity, and selectivity might also turn out to be strikingly advantageous in disease therapeutics (Suvarna et al., 2025).

### 10.2 Future of giardiasis treatment

There have been several attempts to identify novel anti-giardial treatments and targets using phenotypic screening against extensive drug and drug-like chemical libraries. A notable finding among them is identification of a set of structurally atypical kinases with a small gatekeeper residue the inhibition of which resulted in impaired growth, attachment and defects in cytokinesis and shows sensitivity to bumped kinase inhibitors, originally designed for *T. gondii* CDPK1 (Hennessey et al., 2016). Second generation nitroimidazoles, cysteine modifying agents, several phytochemicals, etc. showed promising anti-giardial effects (Argüello-García et al., 2020). An approach to identify protein adducts formed by nitroimidazoles by adductome analysis and multidimensional proteomics obtained over twenty potential drug targets including NADP-specific glutamate dehydrogenase and peroxiredoxin which, when inhibited, exhibited significant reduction in proliferation (Lauwaet et al., 2020). Benchimol et al. (2022) highlighted that unique organellar and cellular structures of *Giardia* and *Trichomonas* (such as the ventral disc, hydrogenosome, mitosomes, ribosomes, and proteasomes) could be promising drug targets for developing more specific and effective antiparasitic therapies, particularly to overcome the limitations and resistance associated with MTZ treatment.

### 10.3 Future of amoebiasis treatment

Current frontline therapy of amoebiasis is largely dependent on MTZ, but treatment with MTZ sometimes comes with undesired consequences like adverse side effects, incomplete eradication of pathogens and emergence of resistance (Ali and Nozaki, 2007). However, *Entamoeba*, as well as *Giardia* and *Trichomonas*, are distinct from higher eukaryotes in having a very specialized sulphur-containing amino acid metabolism which makes them a potent drug target. Ali and Nozaki (2007) particularly highlighted the enzyme methionine γ-lyase, that converts sulphur-containing analogues into toxic products, as a promising candidate. Enzymes of redox homeostasis pathways are also suggested to be promising candidate for drug target considering the parasites’ reliance on these enzymes to withstand oxidative stress (Leitsch et al., 2018). As for new drugs, few hydrazine derivatives of MTZ showed better anti-amoebic effect than MTZ with significant binding affinity with thioredoxin reductase, and a subset of these compounds were lipophilic suggesting the likelihood of better uptake by the parasitic cells (Ansari et al., 2015). Overall, repurposing existing compounds, applying new combination therapies along with finding new targets like redox metabolism, lipid metabolism, should be considered for one health visionary (Santosh and Rebello, 2022).

### 10.4 Future of pylorisis treatment

Increasing global resistance against MTZ calls for the implementation of improved and alternative therapeutic strategies. Inclusion of probiotics/prebiotics, antimicrobial peptides, natural products, bacteriophage-based approaches in alternative therapies as well as improved adjuvant and/or nanoparticle-based targeted drug delivery system are likely to pave effective paths for future-directed strategies (Sousa et al., 2022). Linolenic acid-MTZ complex effectively killed *H. pylori* by inducing oxidative stress, DNA damage leading to phosphatidylserine externalization, cell morphology perturbation etc (Zhou et al., 2023). In the most recent study, carried out by Fiedler et al. (2026), activated MTZ and its ether analogue Metro-P3 were observed to inhibit the HpGroEL, a chaperonin, and a thiol peroxidase HpTpx, indicating their potency as drug targets with Metro-P3 having a stronger binding efficacy and inhibitory effect on the HpTpx. The synergistic effect of drug activation generating oxidative stress and inhibition of the bacterial antioxidant defence results in the increased accumulation of toxic radicals and promotes cell death.

## 11 Discussion

MTZ remains a cornerstone of therapy against anaerobic protozoan pathogenic parasites, *E. histolytica*, *T. vaginalis*, *G. lamblia*, as well as microaerophilic Gram-negative bacteria *H. pylori*, because its antipathogenic activity depends on a biochemical principle, i.e., intracellular reductive activation under low redox conditions that differs markedly from that of mammalian hosts, making their metabolic pathways attractive drug targets. In this review, we have discussed the current understanding of MTZ activation and resistance across clinically significant pathogens and have also attempted to highlight lacunae in this domain, envisioning the engagement of future research in filling those gaps.

Involvement of different pathways/factors in MTZ activation across the organisms discussed here is summarized in **Table 7**, and is ranked depending on direct biochemical evidence (level 1), functional genetic evidence (level 2), correlative observations (level 3), and mechanistic hypotheses (level 4).

**Table 7:** Summary and evidence-based ranking of pathways and factors involved in metronidazole activation across the organisms discussed.

Activation/resistance factor	Organism(s)	Proposed role	Evidence level	Key references
PFOR/Ferredoxin	*T. vaginalis*	Major activator (hydrogenosomal); downregulation correlates with anaerobic resistance	Level 1 (reduction) + Level 3 (resistance association)	Müller (1983); Land et al. (2001, 2004); Crossnoe et al. (2002)
	*G. lamblia*	Primary activator via FdI; FdII/FdIII not coupled	Level 1 (reduction) + Level 3 (resistance association)	Townson et al. (1996); Brown et al. (1998); Leitsch et al. (2011)
	*E. histolytica*	Major activator; resistance not consistently linked to PFOR downregulation	Level 1 (reduction) + Level 3 (resistance association)	Duchêne (2016); Samarawickrema et al. (1997); Penuliar et al. (2015)
	*H. pylori*	Secondary/contributory activator	Level 1 (reduction) + Level 3 (resistance association)	Hoffman et al. (1996); Kaihovaara et al. (1998); van der Wouden et al. (2001)
Malic Enzyme (ME)	*T. vaginalis*	Indirect contributor; supplies NADH and pyruvate to hydrogenosomal electron transport	Level 1 (pathway role, indirect) + Level 3 (resistance association)	Land et al. (2001); Hrdý et al. (2005); Rasoloson et al. (2002)
	*E. histolytica*	Hypothesized alternative activator; direct evidence lacking	Level 3 (expression correlation) + Level 4 (hypothesis)	Chakrabarty et al. (2025)
	*G. lamblia*	Unknown; no evidence reported	Not yet investigated	GiardiaDB (genomic presence only)
Thioredoxin Reductase (TrxR)	*T. vaginalis*	Major cytosolic activator; competes with thioredoxin for electrons	Level 1 (reduction + adducts)	Leitsch et al. (2009)
	*G. lamblia*	Contributory activator; importance uncertain	Level 1 (reduction) + Level 2 (negative functional) + Level 3 (resistance association)	Leitsch et al. (2011, 2016)
	*E. histolytica*	Major cytosolic activator; also a primary drug target	Level 1 (reduction + adducts + functional impairment)	Leitsch et al. (2007); Schlosser et al. (2013)
Nitroreductases (General)	*T. vaginalis*	Ntr4Tv/Ntr6Tv: drug detoxification; SNPs associated with resistance	Level 3 (genetic association) + Level 4 (mechanistic hypothesis)	Paulish-Miller et al. (2014); Graves et al. (2023)
	*G. lamblia*	GlNR1: possible activator; GlNR2: detoxification	Level 2 (functional genetic) + Level 4 (structural inference for GlNR1)	Müller et al. (2007b, 2013, 2015); Nillius et al. (2011)
	*E. histolytica*	Ehntr1/Ehntr2: activators; NIM: detoxification	Level 2 (functional genetic) + Level 3 (genetic association)	Pal et al. (2009)
	*H. pylori*	RdxA: primary activator; FrxA: secondary activator	Level 1 (reduction) + Level 2 (functional genetic)	Sisson et al. (2002); Kwon et al. (2000); Jeong et al. (2000); Kim et al. (2009)
Ntr8Tv (specific)	*T. vaginalis*	Hypothetical activator via iron homeostasis; downregulation linked to resistance	Level 1 (iron reduction) + Level 3 (resistance association) + Level 4 (MTZ activation hypothesis)	Paunkov et al. (2025)
Cysteine/Iron/FR1	*T. vaginalis*	Proposed alternative activation pathway; oxygen-scavenging FR1 facilitates Fe²^+^[cysteine]_2_ complex	Level 2 (complementation) + Level 3 (resistance association) + Level 4 (pathway hypothesis)	Mayr et al. (2024); Leitsch et al. (2014)
Fe-SOD/Peroxiredoxin/Flavin reductase	*E. histolytica*	Antioxidant defence; overexpression confers partial resistance	Level 2 (functional genetic) + Level 3 (resistance association)	Wassmann et al. (1999); Samarawickrema et al. (1997)

In the absence of a licenced vaccine in the market, MTZ remains the cornerstone of therapy for invasive amoebiasis, but unfortunately, important factors like the influence of gut microbiome on *Entamoeba* colonization, invasion, and disease severity, difficulties in distinguishing the *E. histolytica* from non-pathogenic *E. dispar* by regular diagnostics, and most importantly the key determinants of why some individuals remain asymptomatic while others develop invasive disease, are often ignored (Shirley et al., 2026). MTZ induces malic enzyme expression, and its intermediates form adducts with malic enzyme in *E. histolytica* (Chakrabarty et al., 2025), which can be explained as an attempt to replenish the functional intracellular ME abundance since this enzyme performs several catalytic activities essential for the anaerobic protozoan’s viability.

Despite multiple reports on resistance, insufficient data on clinically important mutations are noticeable. Resistance is often multifactorial and, therefore, transcriptional remodelling, metabolic plasticity, altered oxygen handling, or natural genetic variations in multiple redox-associated genes may give rise to a resistance phenotype.

Since the antiparasitic function of nitroimidazoles is evident and well-established to date, evaluating different MTZ derivatives against anaerobic protozoans, especially the compounds that would specifically target or bypass the already proven factors for resistance, might turn out to be beneficial (Suvarna et al., 2025; Jarrad et al., 2016; Dunn et al., 2010; Adagu et al., 2002). The components of the amino acid metabolism pathway are upregulated in the *Trichomonas* resistant strain, indicating a potential contribution to drug susceptibility (Lin et al., 2020). Overexpression of multidrug resistance efflux pumps has also been documented and claims for an in-depth understanding (Huang et al., 2021). Reactive metabolites of MTZ and other nitroimidazoles form covalent adducts with several proteins, including thioredoxin reductase. This is particularly significant because this TrxR-MTZ adduct formation can be viewed as the parasites’ own mechanism to inhibit MTZ reduction further and thereby the synthesis of any more toxic intermediates. As the ultimate objective of this review is to identify any potential drug (metronidazole or any other compound) target candidate protein/pathway that may encourage translational research to design new drugs or metronidazole-derivative compounds, we envisage, based on our understanding, that in hydrogenosome-containing *Trichomonas*, TrxR could be considered as a target for new drugs.Although it has not been explicitly demonstrated that TrxR is the major determinant of metronidazole resistance in *T. vaginalis*, we suggest investigating the comparative quantitative rate of metronidazole activation and the TrxR expression profile to acquire a better understanding.

The metabolism of sulphur-containing amino acids is one of these parasites’ primary metabolic differences with their hosts. Strong therapeutic selectivity results from the absence or significant divergence of sulphur amino acid catabolism and cysteine production in humans (Ali and Nozaki, 2007). Additionally, in *H. pylori*, the disulphide reductase-associated electron flow contributes to MTZ reduction independent of resistance phenotype, though most likely, disulphide reductases are not primary dedicated MTZ reductases, but rather compete with MTZ for intracellular reducing power, thereby modulating the extent of drug activation (Kaakoush and Mendz, 2005). Therefore, the new approaches to drug design should consider manipulating these pathways.

Brown et al. (1998) proposed that the anaerobic metabolism of *G. duodenalis* exhibits a strong evolutionary affinity with the eubacterial metabolism because it relies on bacterial-type enzymes such as PFOR and ferredoxin instead of the mitochondrial pyruvate dehydrogenase complex. The authors suggested that these shared metabolic features could provide insight into the evolution of anaerobic energy metabolism in eukaryotes. However, subsequent phylogenomic analyses have shown that *Giardia* is not a primitive pre-mitochondrial eukaryote but a highly specialized metamonad whose bacterial-like metabolic pathways likely reflect adaptation to anaerobiosis and, in some cases, horizontal gene transfer rather than direct evolutionary ancestry. The precise biochemical function of GlNR1 in metronidazole metabolism remains unresolved. Structural features suggest electron-transfer capability; overexpression confers susceptibility, but direct reduction of metronidazole by purified GlNR1 has not been conclusively demonstrated.

All these protozoan parasites are so primitive that they do not possess a typical mitochondrion, instead, sometimes they possess very specialized structures like hydrogenosome in *Trichomonas* and mitosome in *Entamoeba*. Unfortunately, cell death pathways are not well-explored in *Trichomonas* and *Giardia.* It is important to understand the pathways involved in the execution process of the cell death which may allow us to fine distinguishing of these pathways with higher eukaryotes and their hosts. For *Entamoeba*, two different but interconnecting modes of cell death by MTZ treatment has been reported. Though a thorough investigation on AIF regulatory and translocation mechanism, and finding partner(s) for DNA degradation is required. *Giardia* seems to initiate a regulated death program, but without a dedicated machinery may degenerate into a necrotic phenotype. Since iron is crucial for *Giardia* and actively sourced from the host, metronidazole may induce ferroptosis through oxidative stress, lipid peroxidation, membrane damage, and weakened antioxidant defenses caused by TrxR inhibition. Therefore, studying ferroptosis in *Giardia* could be a new and promising perspective in cell death research.
